# Block copolymer gyroids for nanophotonics: significance of lattice transformations

**DOI:** 10.1515/nanoph-2021-0644

**Published:** 2022-01-18

**Authors:** Haedong Park, Seungyun Jo, Byungsoo Kang, Kahyun Hur, Sang Soon Oh, Du Yeol Ryu, Seungwoo Lee

**Affiliations:** School of Physics and Astronomy, Cardiff University, Cardiff CF24 3AA, UK; Department of Chemical and Biomolecular Engineering, Yonsei University, Seoul 03722, Republic of Korea; KU-KIST Graduate School of Converging Science and Technology, Korea University, Seoul 02841, Republic of Korea; Materials and Life Science Research Division, Korea Institute of Science and Technology, Seoul 02792, Republic of Korea; Department of Integrative Energy Engineering, Department of Biomicrosystem Technology & KU Photonics Center, Korea University, Seoul 02841, Republic of Korea

**Keywords:** block copolymers, chirality, gyroids, optical metamaterials, Weyl points

## Abstract

A gyroid crystal possesses a peculiar structural feature that can be conceptualized as a triply periodic surface with a constant mean curvature of zero. The exotic optical properties such as the photonic bandgap and optical chirality can emerge from this three-dimensional (3D) morphological feature. As such, gyroid crystals have been considered as the promising structures for photonic crystals and optical metamaterials. To date, several methods have been proposed to materialize gyroid crystals, including 3D printing, layer-by-layer stacking, two-photon lithography, interference lithography, and self-assembly. Furthermore, the discovery of Weyl points in gyroid crystals has further stimulated these advancements. Among such methods, the self-assembly of block copolymers (BCPs) is unique, because this soft approach can provide an easy-to-craft gyroid, especially at the nanoscale. The unit-cell scale of a gyroid ranging within 30–300 nm can be effectively addressed by BCP self-assembly, whereas other methods would be challenging to achieve this size range. Therefore, a BCP gyroid has provided a material platform for metamaterials and photonic crystals functioning at optical frequencies. Currently, BCP gyroid nanophotonics is ready to take the next step toward topological photonics beyond the conventional photonic crystals and metamaterials. In particular, the intrinsic lattice transformations occurring during the self-assembly of BCP into a gyroid crystal could promise a compelling advantage for advancing Weyl photonics in the optical regime. Lattice transformations are routinely considered as limitations, but in this review, we argue that it is time to widen the scope of the lattice transformations for the future generation of nanophotonics. Thus, our review provides a comprehensive understanding of the gyroid crystal and its lattice transformations, the relevant optical properties, and the recent progress in BCP gyroid self-assembly.

## 1 Introduction

### 1.1 Historical background

Discovered in 1970 by A. Schoen, a gyroid commonly features a triply periodic surface with a constant mean curvature (CMC) of zero [1]. This unique structural characteristic can endow the peculiar optical properties of matter – from subwavelength-scale optical metamaterials [2], [3], [4], [5], [6], [7], [8], [9], [10] to wavelength-scale photonic crystals [11], [12], [13], [14], [15], [16], [17], [18], [19], [20], [21]. Specifically, over the last two decades, fruitful theoretical [11], [12], [13], [14], [15], [16], [17], [18], [19], [20], [21], [22], [23], [24], [25], [26], [27], [28], [29], [30] and experimental [13, 15], [16], [17, 31], [32], [33], [34], [35], [36] achievements have been reported regarding this topic. However, the importance of gyroids as an optical structure was not well recognized during the 1970–1990s, because a gyroid had rarely been realized, especially at the nanoscale [37], [38], [39].

Owing to its three-dimensional (3D) structural complexity, conventional monolithic lithography is incompatible with gyroid fabrication. Although a macroscopic gyroid (characteristic length scale ranging from millimeters to centimeters) can be readily obtained using common 3D printing [16, 33, 40] or a manual assembly process (e.g., selective drilling of the flat layers and 3D stacking of them [36, 41]), the fabrication of an intermediate nanoscale gyroid (ranging within 200–300 nm) based on such common fabrication methods would be challenging. In context, nanoscale 3D printing (i.e., two-photon lithography [34, 42], [43], [44], [45]) has extended the lower scale limit of the gyroid lattice size to 300–400 nm [42, 44, 46]. However, this process is limited to a single gyroid, which can be applied to only visible photonic crystals [11, 13, 15, 17, 19] and unsuitable for optical metamaterials. Although interference (or holographic) lithography (IL) [24, 47], [48], [49], which could be regarded as a competing method with two-photon lithography, has yielded a single gyroid-like structure with an approximate lattice size of 500 nm [12], it was not a perfect gyroid. Meanwhile, biotemplating was suggested as a bypass method to obtain nanoscale gyroid [13, 14, 17, 25, 31, 32]; however, this method lacks controllability over the lattice size and geometry. Overall, a top-down lithographic definition is still challenging to address submicron-scale (single and double) gyroids with tunable lattice sizes.

In the late 1980s, the bottom-up self-assembly of block copolymer (BCP) paved the unique way for the experimental access to the nanoscale gyroid by Thomas et al. (in 1986) [37]. At the initial stage, the accessible lattice size was limited to a sub-50-nm scale. This scale can be utilized for optical metamaterials [3, 5], whereas the visible photonic crystals [11, 13, 15, 17, 19] and Weyl materials demand a significantly larger scale (100–300 nm) [29, 30]. Recently, the upper limit of this scale has been considerably expanded to approximately 300 nm by increasing the accessible molecular weight of BCP self-assembly for gyroids [50]. Consequently, the BCP self-assembly can bridge the scale gap of the top-down-accessible gyroid and fulfill the scale requirements for “gyroid nanophotonics” – ranging from optical metamaterials to visible photonic crystals/Weyl materials [11, 13, 15, 17, 19, 29, 30]. In addition, the softness of its self-assembly process (i.e., solvent-assisted processes over a large area and subsequent thermal annealing [11, 51], [52], [53]) has promoted the rapid uptake of BCP gyroid as a nanophotonic material pallet for the experts and newcomers alike. Since the 2000s, BCP gyroid nanophotonics has undergone considerable progress [2, 11, 50], [51], [52], [53], [54], [55], [56], including Bragg-diffractive photonic crystals [9] and chiral metamaterials [3, 4, 8], [9], [10, 46, 57, 58]. Inspired by these experimental achievements, Weyl photonic crystals [28, 30, 59] and negative-index optical metamaterials [2, 3, 7] have been theoretically designed under the domain of BCP gyroid.

Nevertheless, currently, the BCP remains an underutilized toolset for nanophotonic scientists and engineers in comparison to conventional lithography. In particular, the research efforts to BCP gyroid nanophotonics have declined, especially in the 2010s, because structural defects such as lattice distortions [60], [61], [62] and grain boundaries (multidomain with dislocations and disclinations) [63] are intrinsically accompanied by BCP self-assembly and hinder the deterministic study of nanophotonics along with their immediate practical usage. Although the epitaxial assembly of BCP (e.g., graphoepitaxy, chemo-epitaxy, etc.) has enabled a large-area, defect-free array [64, 65], this approach has been mainly limited to 2D lines and circle patterns. The epitaxial assembly of a BCP into a single-crystalline 3D gyroid is still challenging over a large-area [64, 65] and is yet to be realized, while the vertical orientation of a BCP gyroid film has been occasionally controlled via interfacial engineering between the BCP and substrate [35, 50], [51], [52, 66], [67], [68].

Equally importantly, the internal lattice transformations of a BCP gyroid [60], [61], [62, 69, 70] had been an out of major scope for a long time; these transformations were merely quantitated two years ago [56]. To date, solvent- or thermal-assisted assembly has been prevalent in the self-assembly of BCP gyroids, as they are considered as an inevitable step for inducing sufficient movement of the polymeric chain. Consequently, these processes cause the BCP film to undergo volumetric expansion during the self-assembly of a gyroid and its subsequent shrinkage. Therefore, the stress primarily occurs along the normal direction, distorting the BCP gyroid lattice [56]. The non-affine rather than affine transformations were found to be induced in the self-assembly of the BCP gyroid. As such, the triclinic lattice of a BCP gyroid is realistically available rather than the cubic lattice [35]. In contrast, most photonic theories regarding double gyroids have been usually founded on cubic unit cells. This gap needs to be addressed. Although, Ref. [35] demonstrated a numerical approach for quantifying the affine transformation across cubic and triclinic cells, the utilization of this theoretical expression cannot be readily amenable to the practically accessible BCP double gyroids. Thus, together with the aforementioned grain boundaries with dislocations and disclinations [60], [61], [62], [63], this lattice transformation could represent a technical challenge that further prevents the deterministic optical study and practical usage of the BCP gyroid.

### 1.2 Scope of review

In this review, we propose that such negatively observable lattice distortions [35, 56] can be a vital aspect of the BCP gyroid, widening the scope of BCP gyroid nanophotonics. The multidomain assemblies of a BCP gyroid are still required to be addressed to enable its deterministic and practical application. However, lattice distortions can act as a pivotal role in topological photonics such as Weyl photonic crystals [30, 35]. Before the advent of Weyl materials [28, 36, 59, 71], the application space of a BCP gyroid was limited to optical metamaterials [3, 4, 6], [7], [8], [9, 46, 57] and photonic crystals [11], [12], [13], [14], [15], [16], [17], [18], [19], [20], [21]. Thus, we organized this review as follows: first, we provided quantitative descriptions of a non-distorted gyroid crystal along with a field guide for the crystal analyses of a gyroid (refer to Section 2). Subsequently, we summarized the numerical approaches to quantify the nanophotonic properties of a dielectric and metallic gyroid. Accordingly, we discussed the theoretical possibility of the nanophotonic characteristics resulting from the crystal structures of a gyroid (refer to Section 3). Thereafter, this theoretical guide was substantiated with a comprehensive overview of state-of-the-art BCP self-assemblies for gyroid structures along its scope of application in conventional nanophotonics (refer to Section 4). To advance to the further phase of the BCP gyroid nanophotonics, we stressed to which the non-distorted gyroid could be affinely and non-affinely transformed based on the currently identified experimental results. In particular, by emphasizing on the current outcomes and subsequently incorporating into numerical calculations, we envisioned that the BCP gyroid, transformed non-affinely, could exhibit a peculiar Weyl point in the photonic crystal lattice (refer to Section 5). Lastly, we suggested possible future research directions by contrasting the obtained numerical predictions in perspective with a realistically accessible experimental approach and potentially competing methods such as IL (refer to Sections 6 and 7).

## 2 Characteristics of gyroid crystals

As discussed earlier in Section 1, a gyroid is a triply periodic surface, i.e., a type of level-set surface [1]. In this section, we initially construct a ball-stick gyroid, denoted as “srs-net” [72] or “skeletal” structure [73], as this approach could facilitate the understanding of gyroid description. Subsequently, we derive its level-set surface using the Fourier expansion.

### 2.1 Ball-stick gyroid

**Figure 1:**
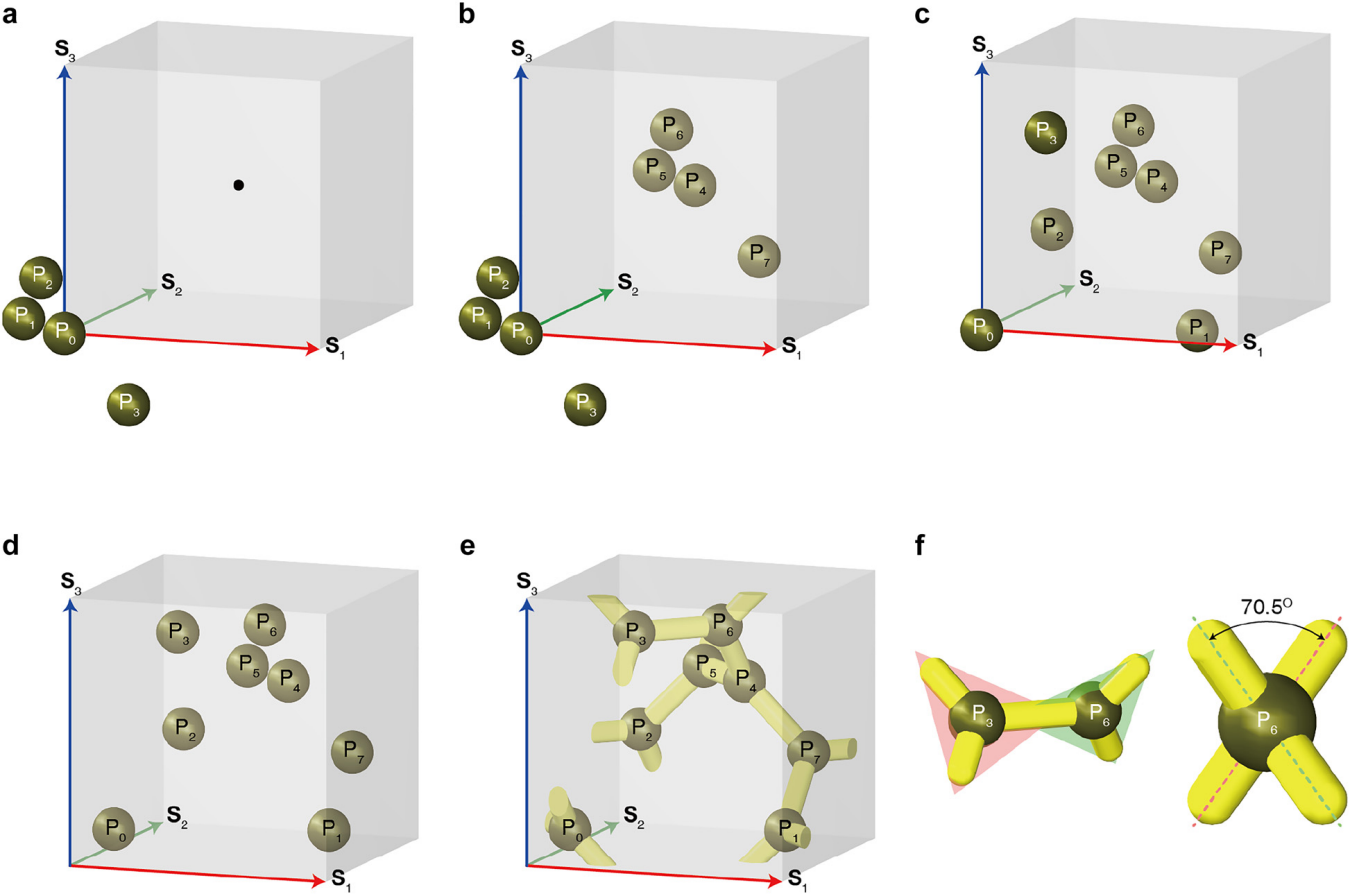
Building a ball-stick gyroid. (a) A BCC lattice and a basis comprising four balls. (b) Additional basis copied from origin to center of cube. (c) Balls only in given cube after considering periodicity. (d) Balls shifted by *a*[1,1,1]/8. (e) Balls and sticks connecting nearest neighboring balls. (f) Dihedral angle by adjacent three-way junctions.

Consider a body-centered cubic (BCC) lattice, where the translation vector(s) of equal magnitude, connecting the first-nearest neighbors, are orthogonal to each other (the array of cubes) [74, 75]. The “BCC lattice” is a special form of the cubic lattice in which an additional lattice point is defined at the center of all the cubes, as depicted by the black point in Figure 1a. Moreover, the lattice vectors **S**_1_ = *a*[1,0,0], **S**_2_ = *a*[0,1,0], and **S**_3_ = *a*[0,0,1] were considered, where *a* denotes a lattice parameter (see Figure 1a).

Subsequently, we placed a ball at [0,0,0] and further added three balls at *a*[−1,1,0]/4, *a*[0,−1,1]/4, and *a*[1,0,−1]/4 (see Figure 1a). These four balls constituted the “basis” group [74], whose elements may occasionally be of various types or shapes. Although the center of this basis was positioned at the origin, its positioning at the origin was not mandatory. More importantly, these bases should be identically placed at every lattice point. As we assumed the BCC lattice, we copied these four balls using (**S**_1_ + **S**_2_ + **S**_3_)/2 (see Figure 1b). Owing to the periodicity of the cube, these two bases were copied along all the translation vectors. Then, we considered only the balls in the cube, and referred them as “crystal structure,” as depicted in Figure 1c. Thus, the crystal structure was formed by placing the basis on every lattice point in a given lattice [74].

For the sake of simplicity, we shifted these eight balls to *a*[1,1,1]/8, as portrayed in Figure 1d. Their coordinates and connectivity are listed in Tables 1 and 2, respectively. As the cube was periodic along the directions of **S**_1_, **S**_2_, and **S**_3_, these balls were equivalently positioned in all the cubes. The minimum distance between any two balls (i.e., distance between the first-nearest neighbors) is $a\sqrt{2}/4$ . Thereafter, we connected these balls with a distance of $a\sqrt{2}/4$ , as illustrated in Figure 1e, and the connections are listed in Table 2. The resultant structure is a notable single gyroid. Overall, the three sticks invariably converged for any given ball point. Therefore, we could visualize a plane defined by the three sticks: the angle between the two planes at two adjacent balls is invariably 70.5288° (see Figure 1f).

**Table 1:** Positions of balls in single gyroid in Figure 1e.

Position	Coordinate	Position	Coordinate
**P**_0_	*a*[1,1,1]/8	**P**_4_	*a*[5,5,5]/8
**P**_1_	*a*[7,3,1]/8	**P**_5_	*a*[3,7,5]/8
**P**_2_	*a*[1,7,3]/8	**P**_6_	*a*[5,3,7]/8
**P**_3_	*a*[3,1,7]/8	**P**_7_	*a*[7,5,3]/8

**Table 2:** Connectivity between balls of single gyroid in Figure 1e.

Connection number	Balls	Connection number	Balls
1	**P**_0_ and **P**_1_−**S**_1_	10	**P**_3_ and **P**_6_
2	**P**_0_ and **P**_3_−**S**_3_	11	**P**_3_ and **P**_0_+**S**_3_
3	**P**_0_ and **P**_2_−**S**_2_	12	**P**_3_ and **P**_5_−**S**_2_
4	**P**_1_ and **P**_6_−**S**_3_	13	**P**_4_ and **P**_5_
5	**P**_1_ and **P**_0_+**S**_1_	14	**P**_4_ and **P**_6_
6	**P**_1_ and **P**_7_	15	**P**_4_ and **P**_7_
7	**P**_2_ and **P**_5_	16	**P**_5_ and **P**_3_+**S**_2_
8	**P**_2_ and **P**_7_−**S**_1_	17	**P**_6_ and **P**_1_+**S**_3_
9	**P**_2_ and **P**_0_+**S**_2_	18	**P**_7_ and **P**_2_+**S**_1_

### 2.2 Standard level-set function of a single gyroid

Although the ball-stick gyroid structure is an easy-to-craft model, advancing our discussions based on this gyroid model would be unsuitable. Thus, we aimed to express a single gyroid structure in a mathematical relation. The mathematical derivations in this subsection are identical to those used in electron-density reconstruction from X-ray scattering analysis [35].

Suppose a function *f*(**X**) is defined at the point **X** = [*X*_1_, *X*_2_, *X*_3_] in the 3D real space, and assume that the gyroid surface corresponds to the set of **X** such that *f*(**X**) = *f*_cut_, where *f*_cut_ is a constant. As our objective is to obtain *f*(**X**) from the ball-stick single gyroid structure, we considered *f*(**X**) as a vector **f**. If the vector **f** can be expressed as $f=\sum_{m}f_{m}{\hat{e}}_{m}$ , then each component of **f** can be expressed with respect to the orthonormal basis ${\hat{e}}_{m}$ as $f_{m}=〈{\hat{e}}_{m}|f〉$ . Similarly, the expansion of function *f*(**X**) elaborates to $f\left(X\right)=\sum_{m}f_{m}e_{m}\left(X\right)$ , where $f_{m}=\int e_{m}^{*}\left(X\right)f\left(X\right)d^{3}X$ is analogous to the inner product of $e_{m}^{*}\left(X\right)$ and *f*(**X**). Additionally, the basis function *e*_*m*_(**X**) was assumed as orthonormal such that $\int e_{m}^{*}\left(X\right)e_{n}\left(X\right)d^{3}X=\delta_{mn}$ . In general, the Legendre polynomials, spherical harmonics, or Bessel functions are examples of orthonormal functions [76].

Herein, we employed a Fourier series that utilized sinusoidal functions as orthonormal functions. Because we deal with periodic structures, sinusoidal functions that also have periodic natures are appropriate. In addition, the sinusoidal functions can be used for affine and non-affine deformations that will be explained later. The function *f*(**X**) is expanded as follows:

(1) $$f\left(X\right)=f_{0}+\underset{m}{\sum }f_{m}^{c}\text{ cos}\left(\frac{2\pi }{a}n_{m}\cdot X\right)+f_{m}^{s}\text{ sin}\left(\frac{2\pi }{a}n_{m}\cdot X\right)\text{,}$$

where the coefficients *f*_0_, $f_{m}^{c}$ , and $f_{m}^{s}$ were evaluated, respectively, as follows:

(2) $$f_{0}=\frac{1}{V}\int_{V}f\left(X\right)d^{3}X\text{,}$$

(3) $$f_{m}^{c}=\frac{2}{V}\int_{V}f\left(X\right)\text{cos}\left(\frac{2\pi }{a}n_{m}\cdot X\right){\text{d}}^{3}X\text{,}$$

(4) $$f_{m}^{s}=\frac{2}{V}\int_{V}f\left(X\right)\text{sin}\left(\frac{2\pi }{a}n_{m}\cdot X\right){\text{d}}^{3}X\text{,}$$

where **n**_*m*_ denotes a three-component vector comprising integers, and *V* = *a* ^3^ denotes the cube volume.

Generally, various calculation methods exist for Eqs. (2)–(4). The first method uses a unit function *f*(**X**), which yields a value of one inside the ball and stick, and otherwise, zero. The implementation of this function involves numerical integrals, and the results of $f_{m}^{c}$ and $f_{m}^{s}$ up to a value of *m* = 28 are listed in Table 3. Accordingly, the integral in Eq. (2) indicates the volume of the ball-stick gyroid structure, and thus, *f*_0_ represents the filling ratio of the ball-stick single gyroid structure to the cube.

**Table 3:** Results of $f_{m}^{c}$ and $f_{m}^{s}$ obtained from numerical integrals on Eqs. (3) and (4); values of $f_{0}$ is 0.02946.

*m*	${\left|n_{m}\right|}^{2}$	n_*m*_	$f_{m}^{c}$	$f_{m}^{s}$
1–3	1	[1,0,0], [0,1,0], [0,0,1]	0	0
4–9	2	[1,1,0], [0,1,1], [1,0,1], [1,−1,0], [0,1,−1], [−1,0,1]	0	0.02785
10–13	3	[1,1,1], [−1,1,1], [1,−1,1], [1,1,−1]	0	0
14–16	4	[2,0,0], [0,2,0], [0,0,2]	0	0
17–28	5	[2,1,0], [0,2,1], [1,0,2], [2,0,1], [1,2,0], [0,1,2]		
		[2,−1,0], [0,2,−1], [−1,0,2], [2,0,−1], [−1,2,0], [0,−1,2]	0	0

For the second method, the function *f*(**X**) was assumed to contain 3D Dirac delta functions, and solely the analytical calculations were required. The Dirac delta function, $\delta^{3}\left(X-P_{n}\right)$ , satisfies the following relation:

(5) $$\delta^{3}\left(X-P_{n}\right)=\left\{ \begin{array}{ll} 0, & X\neq P_{n}\\ +\infty , & X=P_{n} \end{array}\right.\text{,}$$

(6) $$\int_{V}\delta^{3}\left(X-P_{n}\right){\text{d}}^{3}X=1\text{.}$$

For an arbitrary continuous function *F*(**X**), the following integral produces the function value at which the delta function is not zero:

(7) $$\int_{V}\delta^{3}\left(X-P_{n}\right)F\left(X\right){\text{d}}^{3}X=F\left(P_{n}\right)\text{.}$$

Subsequently, the function *f*(**X**) is defined by the summation of eight delta functions whose peaks are located at the ball positions **P**_*n*_ listed in Table 1.

(8) $$f\left(X\right)=\sum_{n=0}^{7}\delta^{3}\left(X-P_{n}\right)$$

Upon substituting this into Eqs. (2) and (3), the following equations were derived, respectively.

(9) $$f_{0}=\frac{1}{V}\int_{V}\left[\overset{7}{\underset{n=0}{\sum }}\delta^{3}\left(X-P_{n}\right)\right]{\text{d}}^{3}X=\frac{1}{V}\overset{7}{\underset{n=0}{\sum }}\int_{V}\left[\delta^{3}\left(X-P_{n}\right)\right]{\text{d}}^{3}X=\frac{8}{V}$$

and

(10) $$\begin{array}{l} f_{m}^{c}=\frac{2}{V}\int_{V}\left[\overset{7}{\underset{n=0}{\sum }}\delta^{3}\left(X-P_{n}\right)\right]\text{cos}\left(\frac{2\pi }{a}n_{m}\cdot X\right){\text{d}}^{3}X\\ \;=\frac{2}{V}\sum_{n=0}^{7}\left[\int_{V}\delta^{3}\left(X-P_{n}\right)\text{cos}\left(\frac{2\pi }{a}n_{m}\cdot X\right){\text{d}}^{3}X\right]\\ \;=\frac{2}{V}\sum_{n=0}^{7}\text{cos}\left(\frac{2\pi }{a}n_{m}\cdot P_{n}\right)\text{.} \end{array}$$

Similarly, Eq. (4) deduced into

(11) $$\begin{array}{l} f_{m}^{s}=\frac{2}{V}\int_{V}\left[\sum_{n=0}^{7}\delta^{3}\left(X-P_{n}\right)\right]\text{sin}\left(\frac{2\pi }{a}n_{m}\cdot X\right){\text{d}}^{3}X\\ \;=\frac{2}{V}\overset{7}{\underset{n=0}{\sum }}\left[\int_{V}\delta^{3}\left(X-P_{n}\right)\text{sin}\left(\frac{2\pi }{a}n_{m}\cdot X\right){\text{d}}^{3}X\right]\\ \;=\frac{2}{V}\overset{7}{\underset{n=0}{\sum }}\text{sin}\left(\frac{2\pi }{a}n_{m}\cdot P_{n}\right)\text{.} \end{array}$$

For each value of *m*, the $f_{m}^{c}$ and $f_{m}^{s}$ reflected the summations for the eight ball positions **P**_*n*_. In addition, the $f_{m}^{c}$ and $f_{m}^{s}$ can be analytically evaluated using the values listed in Table 1 and referring **n**_*m*_ charted in Table 3. Corresponding results up to *m* = 28 are listed in Table 4

**Table 4:** Results of $f_{m}^{c}$ and $f_{m}^{s}$ obtained from Eqs. (10) and (11).

*m*	${\left|n_{m}\right|}^{2}$	n_*m*_	$f_{m}^{c}$	$f_{m}^{s}$
1–3	1	[1,0,0], [0,1,0], [0,0,1]	0	0
4–9	2	[1,1,0], [0,1,1], [1,0,1], [1,−1,0], [0,1,−1], [−1,0,1]	0	8/*V*
10–13	3	[1,1,1], [−1,1,1], [1,−1,1], [1,1,−1]	0	0
14–16	4	[2,0,0], [0,2,0], [0,0,2]	0	0
17–28	5	[2,1,0], [0,2,1], [1,0,2], [2,0,1], [1,2,0], [0,1,2]		
		[2,−1,0], [0,2,−1], [−1,0,2], [2,0,−1], [−1,2,0], [0,−1,2]	0	0

**Figure 2:**
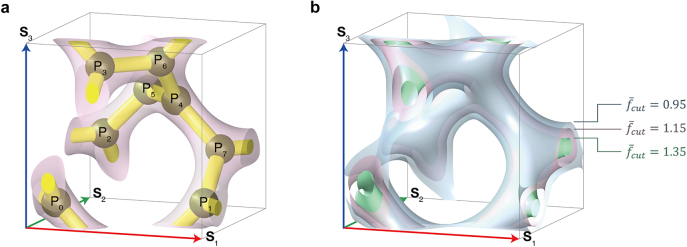
Level-set surface of single gyroid. (a) Level-set surface enclosing ball-stick single gyroid; ${\bar{f}}_{\text{cut}}=1.15$ . (b) Single gyroid level-set surfaces with various values of ${\bar{f}}_{\text{cut}}$ .

The results of both the methods indicated that only $f_{m}^{s}$ for ${\left|n_{m}\right|}^{2}=2$ and *f*_0_ yielded a nonzero value, whereas those of the rest were zero. Thus, we can derive the following formula based on Eq. (1) by accumulating these low-order terms:

(12) $$\begin{array}{l} f\left(X\right)=f_{0}+f_{<110>}^{s}[\text{sin}\left\{\frac{2\pi }{a}\left(X_{1}+X_{2}\right)\right\}+\text{sin}\left\{\frac{2\pi }{a}\left(X_{2}+X_{3}\right)\right\}+\text{sin}\left\{\frac{2\pi }{a}\left(X_{3}+X_{1}\right)\right\}\\ +\text{sin}\left\{\frac{2\pi }{a}\left(X_{1}-X_{2}\right)\right\}+\text{sin}\left\{\frac{2\pi }{a}\left(X_{2}-X_{3}\right)\right\}+\text{sin}\left\{\frac{2\pi }{a}\left(X_{3}-X_{1}\right)\right\}]\text{,} \end{array}$$

where $f_{<110>}^{s}$ denotes the value of $f_{m}^{s}$ for ${\left|n_{m}\right|}^{2}=2$ . As this is a function defined in the 3D space, the set of **X** satisfying *f*(**X**) = *f*_cut_ formed a curved surface, and the set of **X** such that *f*(**X**) > *f*_cut_ or *f*(**X**) < *f*_cut_ indicated a bulk region. The above equation can be simplified as follows:

(13) $$\begin{array}{l} f_{\text{SG}}\left(X\right)=\frac{1}{2}[\text{sin}\left\{\frac{2\pi }{a}\left(X_{1}+X_{2}\right)\right\}+\text{sin}\left\{\frac{2\pi }{a}\left(X_{2}+X_{3}\right)\right\}+\text{sin}\left\{\frac{2\pi }{a}\left(X_{3}+X_{1}\right)\right\}\\ \;+\text{sin}\left\{\frac{2\pi }{a}\left(X_{1}-X_{2}\right)\right\}+\text{sin}\left\{\frac{2\pi }{a}\left(X_{2}-X_{3}\right)\right\}+\text{sin}\left\{\frac{2\pi }{a}\left(X_{3}-X_{1}\right)\right\}]\text{,} \end{array}$$

and the surface and bulk regions can be expressed as $f_{\text{SG}}\left(X\right)={\bar{f}}_{\text{cut}}$ and $f_{\text{SG}}\left(X\right)>{\bar{f}}_{\text{cut}}>0$ , respectively. The alternative expression of Eq. (13) based on the relationship: (1/2)[sin{(*A* + *B*)/2} + sin{(*A* − *B*)/2}] = sin *A* cos *B* can be considered as the notable level-set function of a single gyroid [28, 77]. For an appropriate ${\bar{f}}_{\text{cut}}$ , the set of **X** satisfying $f_{\text{SG}}\left(X\right)={\bar{f}}_{\text{cut}}$ emerges as a surface that approximately wraps the ball-stick single-gyroid structure (see Figure 2a). Moreover, various surfaces can be generated using several ${\bar{f}}_{\text{cut}}$ , and three examples are shown in Figure 2b.

The *f*_SG_(**X**) is defined in Eq. (13) as the standard level-set surface of a single gyroid. This quantification of the BCC gyroid acts as the foundation for discussions on affine and non-affine deformations, which are further detailed in Section 5.

### 2.3 Double gyroid

**Figure 3:**
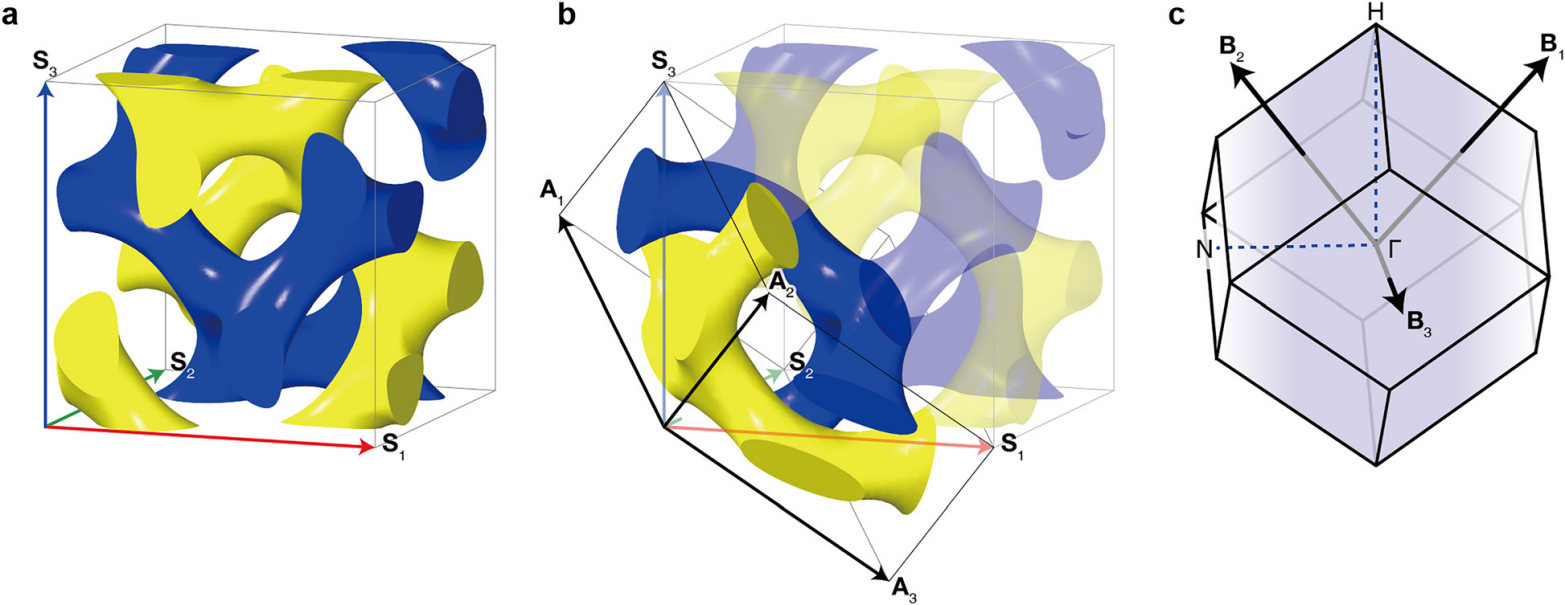
(a) and (b) Double gyroids in conventional and primitive cells, respectively. (c) First Brillouin zone of BCC lattice. High symmetric directions marked here are ΓN = (1/2)(−**B**_1_ + **B**_2_) and ΓH = (1/2)(**B**_1_ + **B**_2_ − **B**_3_).

Regarding Eq. (13), we can consider a surface with *f*_SG_(**X**) = 0. In addition, we constituted a double gyroid by placing two single gyroids $f_{\text{SG}}\left(X\right)>{\bar{f}}_{\text{cut,}1}>0$ and $f_{\text{SG}}\left(X\right)<{\bar{f}}_{\text{cut},2}<0$ in the same space, as depicted in Figure 3a. These two single gyroids do not intersect with each other, and they exist on one side as well as the other side of the surface, *f*_SG_(**X**) = 0. More precisely, this double gyroid exhibited inversion symmetry for identical volumes of the two single gyroids, i.e., ${\bar{f}}_{\text{cut,}1}={\bar{f}}_{\text{cut,}2}$ , which is in contrast to that of the single gyroid.

In context, any structure containing two non-intersecting single gyroids is called a double gyroid. Note that the two gyroids in the double gyroid did not exhibit any translation symmetry. Thus, we can use the same unit cell for both the single and double gyroid crystals, if their translational circumstances are identical. This is one of the major differences as compared to a diamond structure; in case two identical single diamond structures are combined to form a double diamond structure, the two constituting single diamonds can be in translational symmetry, and the double diamond can be described by a simple cubic lattice instead of a face-centered-cubic (FCC) cell [78, 79].

Thus, two mathematical methods were used to describe the double gyroid. The first constitutes a new relation (distinct to Eq. (13)) that could express all the level-set surfaces for both the single gyroids in a double gyroid [77, 80]. The alternative method is to prepare two similar or same formula *f*_SG_(**X**) for the given two single gyroids [30].

### 2.4 Primitive and conventional unit cells

In the previous two sections, the discussion concerned a cube that was periodically placed along the **S**_1_, **S**_2_, and **S**_3_-directions. As such, a unit that repeats along the vectors defining the lattice is called a “unit cell.” There are several selections for setting a unit cell. In particular, we would address the conventional and primitive cells, because certain studies used conventional cells, whereas others used primitive cells; thus, we explain all these cases.

A unit cell in the BCC lattice is frequently selected as a cube as the last “C” in “BCC” refers to “cubic,” which is the so-called conventional cell. The lattice vectors **S**_1_, **S**_2_, and **S**_3_ were set along the three adjacent edges (Figure 3a). They were orthogonal to each other (**S**_*i*_⋅**S**_*j*_ = *δ*_*ij*_), and their magnitudes were the same (|**S**_1_| = |**S**_2_| = |**S**_3_| = *a*). Although, many cases of the Cartesian coordinate system use the same directions of the vectors as that of the lattice vectors, this consideration is not mandatory. Regardless of the Cartesian coordinate system used, the lattice vector magnitudes do not vary, and the conventional cell volume *V* is set as *a* ^3^. Moreover, the lattice points were positioned at the center and eight vertices of the conventional cell. In case the eight adjacent conventional cells shared a vertex, the lattice point at each vertex was counted as only 1/8. Thus, a single BCC conventional cell has two lattice points within which repeatability exists. Thus, the detailed perspective at a lattice point is identical to that of the other lattice point (see Figures 2 and 3a).

A single gyroid in a conventional cell has eight three-way junctions, as illustrated in Figures 1e and 2. In particular, two connections passed via a single face of the cell, where each connection linked the two adjacent three-way junctions. In case of a double gyroid, a single face has 4 connections.

If this gyroid is under mechanical stress, the lattice fidelity of the unit cell is deformed in an affine or non-affine manner. Unless the deformation is hydrostatic, the deformed lattice is not a cube, and thus, “BCC” cannot be used any more (e.g., affine deformation). However, it remains a parallelepiped with equal number of faces, edges, and vertices as the cubic cell. Therefore, the geometry of a BCP double gyroid can still be described, even if the conventional cell is generally a non-cube. This description requires simple mathematical adjustment, which is discussed in Section 5.

Furthermore, we can define a unit cell enclosing only a single lattice point, which is called a “primitive cell.” One of the primitive cells in the BCC lattice system was obtained by connecting the nearest-neighbor lattice points, as depicted in Figure 3b. The eight lattice points were located at the vertices of the parallelepiped, and eight adjacent parallelepipeds shared a single lattice point. Therefore, the total number of lattice points in the primitive cell was one, and the parallelepiped cells could be periodically copied along the direction of the lattice vectors. The lattice vectors **A**_1_, **A**_2_, and **A**_3_ can be expressed with respect to **S**_1_, **S**_2_, and **S**_3_ as

(14) $$A_{1}=\frac{1}{2}\left(-S_{1}+S_{2}+S_{3}\right)$$

(15) $$A_{2}=\frac{1}{2}\left(S_{1}-S_{2}+S_{3}\right)$$

(16) $$A_{3}=\frac{1}{2}\left(S_{1}+S_{2}-S_{3}\right)$$

and, inversely, **S**_1_, **S**_2_, and **S**_3_ with respect to **A**_1_, **A**_2_, and **A**_3_ as

(17) $$S_{1}=A_{2}+A_{3}$$

(18) $$S_{2}=A_{3}+A_{1}$$

(19) $$S_{3}=A_{1}+A_{2}.$$

The simpler expressions include $A_{i}=-S_{i}+\sum_{j=1}^{3}S_{j}/2$ and $S_{i}=-A_{i}+\sum_{j=1}^{3}A_{j}$ , respectively. If we apply **S**_1_ = *a*[1,0,0], **S**_2_ = *a*[0,1,0], and **S**_3_ = *a*[0,0,1] as used in Section 2.1, the lattice vectors of the primitive cell can be expressed as **A**_1_ = (*a*/2)[−1,1,1], **A**_2_ = (*a*/2)[1,−1,1], and **A**_3_ = (*a*/2)[1,1,−1]. If an alternative coordinate system is used, these expressions may vary. However, their magnitudes are commonly fixed at $a\sqrt{3}/2$ . The angle between any two lattice vectors is 109.4712°, regardless of the coordinate system. The volume of the parallelepiped defined by these lattice vectors is *V*_**A**_ = **A**_1_⋅(**A**_2_ × **A**_3_) = *a* ^3^/2 = *V*/2, which denotes the smallest volume that a unit cell can occupy in the BCC lattice system. As depicted in Figure 3b, a single gyroid in a BCC primitive cell possesses four three-way junctions. The number of connections of the single (double) gyroid passing a single surface of the primitive cell is one (two).

### 2.5 Wigner–Seitz cell and first Brillouin zone

The Wigner–Seitz cell is an additional type of BCC primitive cell, which is defined as follows: first, for a lattice point in a 3D space, we can connect the lattice point with its neighboring lattice points by drawing lines. Then, the planes normal to each line were placed at the midpoints of each line. The smallest space by the planes is the Wigner–Seitz cell. As discussed in the previous section, the Wigner–Seitz cell contains only one lattice point, similar to the primitive cell. Furthermore, the two cells occupied equal volumes.

In case the structure is deformed (i.e., its conventional cell becomes non-cube), the number of faces, edges, and vertices of the primitive cell were set as constant, and only the lattice vectors varied. In contrast, these quantities of the Wigner–Seitz cell can become completely distinct, even for a small deformation. Thus, the primitive cell is frequently used to describe the structure in real space.

In the reciprocal space, the Wigner–Seitz cell, referred to as the “first Brillouin zone” or simply “Brillouin zone,” is prevalently used for the quantitation of photonic band structures. For the primitive cell in Figure 3b, the reciprocal lattice vectors **B**_1_, **B**_2_, and **B**_3_ can be expressed as

(20) $$B_{1}=2\pi \frac{A_{2}\times A_{3}}{A_{1}\cdot \left(A_{2}\times A_{3}\right)}$$

(21) $$B_{2}=2\pi \frac{A_{3}\times A_{1}}{A_{1}\cdot \left(A_{2}\times A_{3}\right)}$$

(22) $$B_{3}=2\pi \frac{A_{1}\times A_{2}}{A_{1}\cdot \left(A_{2}\times A_{3}\right)}\text{.}$$

The Brillouin zone of the BCC lattice can be quantified according to the definition of the Wigner–Seitz cell, as depicted in Figure 3c. The high-symmetry points are denoted as ΓN = (1/2)(−**B**_1_ + **B**_2_) and ΓH = (1/2)(**B**_1_ + **B**_2_ − **B**_3_).

## 3 Nanophotonic characterizations and characteristics of gyroid

### 3.1 Modeling photonic band structures

Recent advances in numerical tools allow the simulation of the optical properties of gyroids with various materials and sizes. However, the accurate prediction and complete understanding of the optical properties of gyroid is challenging, and high-accuracy simulations still require a large amount of memory and simulation time. This is because the gyroid structure is a complex network, and the optical properties of the materials depend on the wavelength. Therefore, several approaches have been developed to evaluate the effective index and photonic band structures for various spectral regimes and different materials. Here, we review the numerical methods and three analytical models that are commonly used for dielectric and metallic gyroids, primarily at optical wavelengths.

As a dielectric gyroid can be considered as a 3D photonic crystal [81], we can consider a photonic band structure, which constitutes the frequency versus wavevector relation along the symmetry lines connecting the symmetry points of the crystal lattice. Based on numerically calculated photonic band structures, we can determine a region of the photonic bandgap, where no propagating light mode is allowed [8]. In prior research, photonic-band calculations of the dielectric gyroid structures are based on the plane-wave expansion method. For instance, Maldovan et al. determined the photonic band structure of dielectric single/double gyroids for the first time and reported that the photonic bandgap appeared for a single gyroid and can be tuned by varying the volume fraction [21].

The plane-wave expansion method is a numerical method that solves the eigenvalue equations derived from the Maxwell’s equations, where the electric fields or dielectric fields are expanded as a summation of plane waves [82]. At the early stages of photonic crystal research in the 1990s, homemade codes were used following the study of Ho et al. [41, 82]. Currently, the MIT photonic band [83] and COMSOL Multiphysics [84] are the most popular software for evaluating the photonic band structures of dielectric gyroid structures.

The plane-wave expansion method is not beneficial for the metallic gyroids, because the metal has intrinsic losses at the optical wavelengths and becomes a perfect electric conductor at longer wavelengths (e.g., terahertz and microwave frequencies). Therefore, the development of numerical methods is essential for accurately accounting the metallic losses using complex dielectric constants. In addition, the dispersion of metals is required to be considered to visualize an accurate photonic band structure. In context, a Drude or Drude–Lorentzian model can be employed to describe the dispersion relation. Moreover, the experimentally measured data by Johnson and Christy [85] or Palik [86] are commonly used. As there is no simple method to analytically evaluate the photonic modes or conduct a simple matrix diagonalization, tools such as COMSOL Physics or Lumerical FDTD Solutions [87] are commonly used for photonic band calculations.

Although the photonic band structure can be calculated using numerical tools, the numerical results do not provide clear insights of the interaction of light with gyroid structures. This is because the photonic mode propagations and transmission/reflection spectra are caused by complicated interactions between the electric and magnetic fields as well as the nonuniform arrangement of materials. Therefore, a simple and intuitive model describing the light propagation in connection to the material and geometrical parameters is required to interpret the numerically obtained photonic band structures.

### 3.2 Effective medium theory

The effective medium theory is perhaps the simplest analytical method that can be used to analyze the optical response of composite materials. Dolan et al. applied both the Maxwell–Garnett theory and Bruggerman theory to predict the effective refractive index of a dielectric gyroid and tested the accuracy of the models by comparing the reflection spectra obtained by numerical simulations and analytical calculations with effective permittivity [5].

In Maxwell–Garnett theory [88, 89], a metallic single gyroid is modeled as an array of metallic spheres, and the effective permittivity *ϵ*_eff_(*ω*) is calculated by solving the following equation:

(23) $$\frac{\left(\epsilon_{1}\left(\omega \right)-\epsilon_{2}\left(\omega \right)\right)f_{1}}{\epsilon_{2}\left(\omega \right)+\left(\epsilon_{1}\left(\omega \right)-\epsilon_{2}\left(\omega \right)\right)A}+\frac{\epsilon_{2}\left(\omega \right)-\epsilon_{\text{eff}}\left(\omega \right)}{\epsilon_{2}\left(\omega \right)+\left(\epsilon_{\text{eff}}\left(\omega \right)-\epsilon_{2}\left(\omega \right)\right)A}=0\text{,}$$

where *ϵ*_1_(*ω*) and *ϵ*_2_(*ω*) denote the permittivity of the first (inclusion) and second (host), components, respectively; *f*_1_ represents the filling fraction of the first component, *A* denotes the depolarization parameter (1/3 for spherical inclusions), and *ω* denotes the optical frequency.

In Bruggerman’s theory [90], a similar expression can be derived for an array of metallic spheres, as expressed below.

(24) $$\frac{\left(\epsilon_{1}\left(\omega \right)-\epsilon_{\text{eff}}\left(\omega \right)\right)f_{1}}{\epsilon_{\text{eff}}\left(\omega \right)+\left(\epsilon_{1}\left(\omega \right)-\epsilon_{\text{eff}}\left(\omega \right)\right)A}+\frac{\left(\epsilon_{2}\left(\omega \right)-\epsilon_{\text{eff}}\left(\omega \right)\right)f_{2}}{\epsilon_{\text{eff}}\left(\omega \right)+\left(\epsilon_{2}\left(\omega \right)-\epsilon_{\text{eff}}\left(\omega \right)\right)A}=0\text{,}$$

where *f*_1_ denotes the filling fraction of the first component. Moreover, this expression can be revised for an array of metallic ellipsoids to improve the model accuracy. In context, Dolan et al. [5] concluded that the three distinct effective-medium approaches provide an appropriate quantitative agreement with the numerical simulations, and the approach based on the Bruggermann theory with metallic ellipsoids displays the most appropriate agreement.

### 3.3 Tri-helical metamaterial model for metallic gyroid

**Figure 4:**
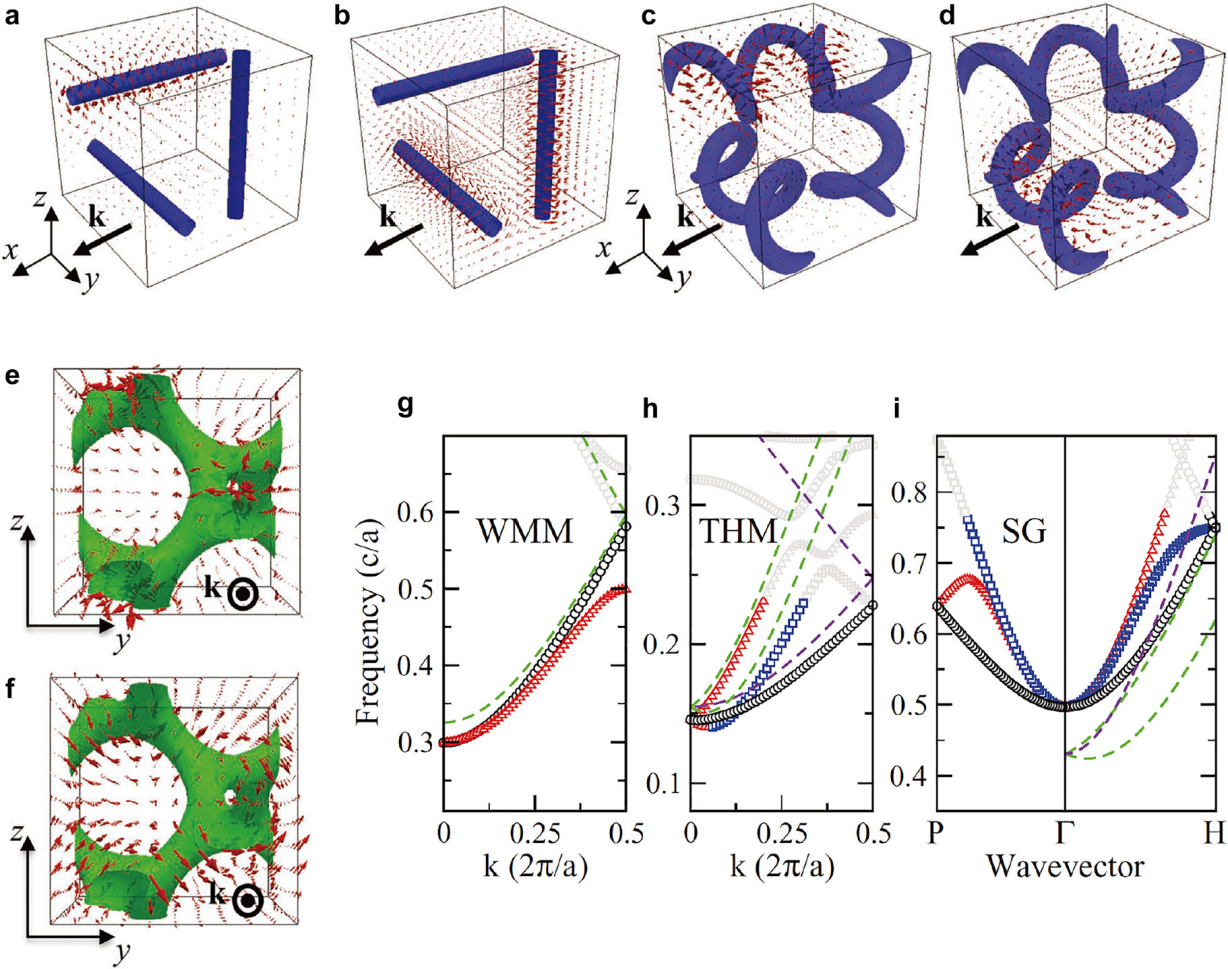
Wire mesh metamaterial (WMM), trihelical metamaterial (THM), and a single gyroid (SG) comprising perfect electric conductor. Electric field vectors for (a) longitudinal and (b) transverse modes in WMM, (c) longitudinal and (d) transverse modes in THM, and (e) longitudinal and (f) transverse modes in SG. (g), (h) and (i) Numerically calculated photonic bands (circles: longitudinal modes, squares, and triangles: transverse modes and analytical photonic bands (dashed lines) for WMM, THM and SG). Reproduced with permission from ref. [7]. Copyright 2013, Wiley-VCH Verlag GmbH Weinheim.

The tri-helical metamaterial (THM) model (Figure 4a–d) can be applied to metallic gyroids from the microwave range to optical wavelengths, and can provide an insight into the origin of the optical chirality with an analytical formula for the dispersion relation [6, 7]. This is an adequate approximation for the photonic bands, particularly close to the Γ point displaying the splitting of a longitudinal mode (Figure 4e) and two transverse modes (Figure 4f), as displayed in Figure 4g–h and relatively accurate for the microwave range. However, it significantly deviates from the numerically calculated band structures at optical wavelengths owing to the plasmonic response, which causes the electric fields to smear into the metallic parts, thereby violating the assumptions related to a perfect electric conductor.

The concept of the THM model was inspired by the wire-mesh metamaterials (WMMs, shown in Figure 4a) proposed by J. B. Pendry in 1998 [91]. The WMM can be modeled as an effective medium with the Drude model with effective plasma frequencies, which can be expressed using geometric parameters such as the periodicity and diameter of the wires. Thereafter, we can approximate a 3D periodic WMM that contains 2D wire-mesh arrays entirely in 3D. The unit cell of the WMM is illustrated in Figure 4a–b. When there is no interaction between the 2D wire-mesh arrays for each direction, we can use the same equations for WMMs that correspond to the two transverse modes of the system. In addition, a longitudinal mode propagates in the axial direction of the wires (as depicted by the electric field in Figure 4a), and this expression can be calculated using effective medium theory. To introduce chirality, we reconfigured all the straight wires to helices with the same chirality, thereby yielding the THM, as presented in Figure 4c–d. Based on this reconfiguration, we induced a coupling between the electric and magnetic fields, contributing toward the chiral response of the optical waves. Upon solving the Maxwell’s equations for the THM geometry, we obtained the expression denoting the three bands for the longitudinal and transverse modes (Figure 4h). This can be compared with the numerical simulations, showing a good agreement at microwave frequencies

The photonic bands obtained by the THM displayed a relatively adequate agreement with the numerically calculated photonic bands of a metallic single gyroid, but it overestimated the optical chirality [7]. The absence of optical chirality in metallic SGs originated because of the identical slopes of the two transverse modes for SG, whereas the slopes varied for THM. This is because the gyroid is a single network, whereas the THM is a collection of three arrays of helices oriented in multiple directions. Therefore, the THM does not consider the electric current flowing from a helix to neighboring helices, and this current act as the primary source of weak chirality in a single plasmonic gyroid.

### 3.4 Double-network metamaterial model

A double-network metamaterial model has been recently studied to consider the current flows between the metallic wires in a metamaterial [92]. This model considers two different networks with opposing electric current flows. Recently, a theoretical approach has been developed to model interlaced metallic wire meshes for explaining light-tunneling anomalies [93]. Then, a novel metamaterial based on connected metallic meshes was proposed to achieve wide-band negative reflection [94]. Evidently, these models surpassed the limit of resonance description of the optical response of metamaterials and facilitated the accurate analysis of complex metallic networks. However, they cannot be directly applied to gyroid structures, because the metamaterials considered in the literature are wire-mesh metamaterials working at microwave frequencies. Much prior to these studies, Hur et al. [2] explained that the electric current in the double gyroid is conserved owing to the current flowing in the opposite direction, which will be detailed in Section 3.6. Although it is challenging to find an extremely accurate analytical expression for the metallic gyroids, this double-network approach is promising as a metallic double gyroid can be modeled with the same topology. Furthermore, it can be applied to a single gyroid as it can account for the connections between the helices, which was ignored in the THM model.

### 3.5 Optical chirality

In geometry, the term “chirality” means the lack of mirror symmetry. Therefore, a chiral structure cannot be mapped onto its mirror images by applying translational and rotational transformations [95]. Moreover, we know that geometrical chirality results in optical chirality, i.e., the asymmetry in transmission and absorption between the left- and right-handed circularly polarized light [96]. Owing to its unique chiral geometry, a gyroid structure exhibits optical chirality, and numerous studies on the optical properties of gyroid structures have focused on the circular dichroism [3, 4, 8], [9], [10]. In context, a chiral beam splitter has been demonstrated at optical wavelengths by utilizing the circular dichroism of the gyroid structure [57].

An additional interesting property originating from the optical chirality is the negative refraction. In 2004, J. B. Pendry theoretically determined that the optical chirality can result in negative refraction in two transverse modes [97]. This study has inspired many researchers in the field of metamaterials, which enabled the search for materials with unusual negative refraction, and the gyroid structure was one of the candidates. However, the chirality of a metallic single gyroid is inadequately weak for creating negative refraction, because the current flows between various helices, as can be observed in Figure 4i [7]. On the contrary, a negative refractive index was theoretically predicted in a metallic double gyroid [2], but the origin is different from that proposed by J. B. Pendry. The details are discussed in the following subsection.

### 3.6 Negative refraction of gyroid metamaterials

**Figure 5:**
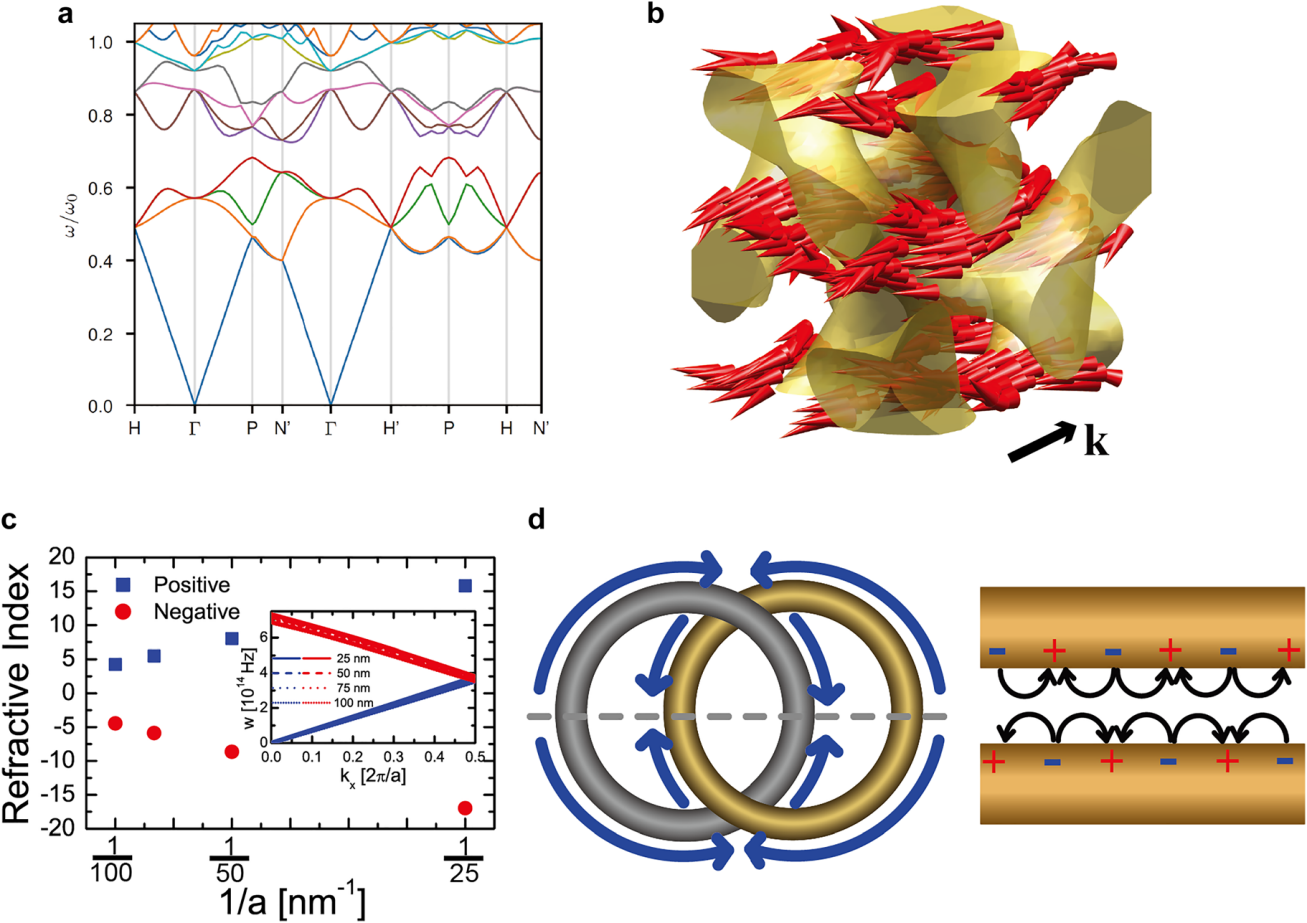
(a) Photonic band structure of double gyroid metamaterial. (b) Negative refraction of double gyroid metamaterial, where red arrows represent photonic energy flux, and **k**-vector represents wave momentum. (c) Refractive index variation as a function of lattice dimension, *a*. (d) Schematic of plasmon resonances to satisfy the electric current conservation. Reproduced with permission from ref. [29]. Copyright 2018, National Academy of Sciences (a); reproduced with permission from ref. [2]. Copyright 2011, Wiley-VCH Verlag GmbH Weinheim (b)–(d).

Previously, photonic band structures of metallic gyroid networks, i.e., gyroid metamaterials, have been computationally investigated [2, 29]. Single and double gyroid metamaterials were found to exhibit distinct photonic responses. The double gyroid metamaterials completely made of a metal (i.e., gold (Au)) can exhibit a low-frequency linear dispersion band, as presented in Figure 5a, whereas the single gyroid metamaterials possess a metallic band gap [2]. In particular, the formation of metallic band gaps in a single gyroid metamaterial can be rationalized using Ampere’s law in an integral form [98]:

(25) $$\oint_{\partial S}B\cdot \text{d}l=\mu_{0}\iint_{S}\left(J+\epsilon_{\infty }\frac{\partial E}{\partial t}\right)\cdot \text{d}A\text{,}$$

where **B** denotes the magnetic field, **E** represents the electric field, and **J** denotes the electric current flux moving across the surface, *S*. Furthermore, the integrals can be discretized to obtain

(26) $$\oint_{\partial S}B\cdot \text{d}l\approx an_{x}\left({\bar{B}}_{x}^{y=y_{0}}+{\bar{B}}_{x}^{y=y_{0}+n_{y}a}\right)+an_{y}\left({\bar{B}}_{y}^{x=x_{0}}+{\bar{B}}_{y}^{x=x_{0}+n_{x}a}\right)\text{,}$$

and

(27) $$\mu_{0}\iint_{S}\left(J+\epsilon_{\infty }\frac{\partial E}{\partial t}\right)\cdot \text{d}A\approx a^{2}n_{x}n_{y}\mu_{0}\left({\bar{J}}_{z}+\epsilon_{\infty }{\bar{\dot{E}}}_{z}\right)$$

owing to the cancellation of integrals inside the metamaterials, where the bars represent the spatially averaged fields; *a*, *n*_*x*_ and *n*_*y*_ denote the lattice dimension, the number of unit cells along *x*, and the number of unit cells along *y*, respectively. Assuming *n*_*x*_ = *n*_*y*_ = *n*, the equality combining Eqs. (25)–(27) becomes

(28) $$\lim_{n\to \infty }an\left({\bar{B}}_{x}^{y=y_{0}}+{\bar{B}}_{x}^{y=y_{0}+n_{y}a}+{\bar{B}}_{y}^{x=x_{0}}+{\bar{B}}_{y}^{x=x_{0}+n_{x}a}\right)\approx \lim_{n\to \infty }a^{2}n^{2}\mu_{0}\left({\bar{J}}_{z}+\epsilon_{\infty }{\bar{\dot{E}}}_{z}\right)\text{.}$$

The separate scaling of both sides of the equation with *n* requires both the sides to be zero. Otherwise, the right-hand side of the equation would grow more rapidly than the left side, resulting in an unphysical infinite magnetic field. At low frequencies, much smaller than the plasma frequency (*ω* << *ω*_*p*_), the electric current flux *J*_*z*_ on the right-hand side of Eq. (28) cannot be negated because $\epsilon_{\infty }{\bar{\dot{E}}}_{z}\propto -\left(\omega^{2}/\omega_{p}^{2}\right){\bar{J}}_{z}\ll {\bar{J}}_{z}$ for Drude metals.

However, in case a counter electric current exists on the other network, the overall electric current can be negated to zero. Therefore, a coupled plasmon resonance of two or more networks is essential for metamaterials, and low-frequency bands of 3D continuous metamaterials require two or more periodic networks to form an active band and satisfy the electric current conservation rule. Shin et al. reported that (*N* − 1) linear dispersion bands exist in *N*-independent continuous network metamaterials [99].

As such, coupled plasmon resonances cannot be obtained and a metallic band gap at low frequencies is expected for single gyroid metamaterials. However, the electric current flux *J*_*z*_ can be cancelled by ${\bar{\dot{E}}}_{z}$ at high frequencies, comparable to the plasma frequency. This is because $\epsilon_{\infty }{\bar{\dot{E}}}_{z}\approx {\bar{J}}_{z}$ and active photonic bands appeared even for single network metamaterials. Thus, the plasma frequency of a metal fundamentally determines the metallic band gap range, as *ω* should be proximate to *ω*_*p*_ for satisfying $\epsilon_{\infty }{\bar{\dot{E}}}_{z}+\left(\omega^{2}/\omega_{p}^{2}\right){\bar{J}}_{z}=0$ .

Double gyroid metamaterials comprise two independent interweaving networks and display one low-frequency linear dispersion band (note that 3D photonic crystals exhibit two low-frequency linear dispersion bands). Moreover, the linear dispersion band becomes a negative refraction band owing to the band folding at the Brillouin zone boundaries (see Figure 5b, where the directions of photonic energy flux and wave momentum are opposite to each other). Theoretically, a variety of photonic crystals exhibit similar negative refractions due to photonic wave interference [100]; negative refraction is not a unique phenomenon, which can be observed only in a plasmonic metamaterial. Nonetheless, plasmons are photonic energy carriers in 3D continuous metamaterials, and in comparison to photons, plasmons can be highly miniaturized to surpass the limitations of photonic crystals such as wavelength commensurability with lattice dimensions. As reported earlier [29], the unit cell size of the BCP-derived materials is usually limited, and their photonic crystal applications are challenging in the visible range. Meanwhile, BCP-derived metamaterials pose no such limitations and can be used in visible-range applications. Furthermore, the refractive index can be controlled by the unit cell dimensions (Figure 5c). This is because the plasmon propagation is primarily governed by the gap distance of the interweaving networks that form a metal–insulator–metal (MIM) waveguide (Figure 5d). Typically, BCP-derived continuous metamaterials contain gaps of several nanometers or tens of nanometers between the networks and exhibit a large positive or negative refractive index.

## 4 Experimental efforts for BCP gyroid nanophotonics

### 4.1 Historical benchmarks of BCP gyroids

More than two decades ago, a well-developed bicontinuous network structure was initially created from multi-arm star BCPs by Thomas et al. [37], and later, they reevaluated this nontraditional structure as a double gyroid structure [38, 39]. Since the discovery of the labyrinthine architecture, the gyroid structures from the BCP self-assembly have enabled the materialization of interconnected 3D networks containing two alternating channels. Bates et al. reported that the gyroid structure could be epitaxially developed from self-assembled hexagonal cylinders as an order-to-order transition in BCP melts [64]; this observation was later substantiated by Matsen’s theoretical approach [65]. Although the BCP gyroid structures were composition-sensitive, they were omnipresent in multi-arm BCPs, diBCPs, and ABA- and ABC-type triBCPs [101], [102], [103]. Specifically, in the ABC-type poly(isoprene-*b*-styrene-*b*-ethylene oxide) (PI-*b*-PS-*b*-PEO), the triBCP self-assembles into alternating A and C domains, thereby producing two distinct channels in a B matrix [103]. For a given gyroid structure with two distinct opposing channels, this approach provides another opportunity to prepare a single gyroid structure in case of selectively removing a single channel.

### 4.2 Material substitutions for BCP gyroid optical metamaterials

**Figure 6:**
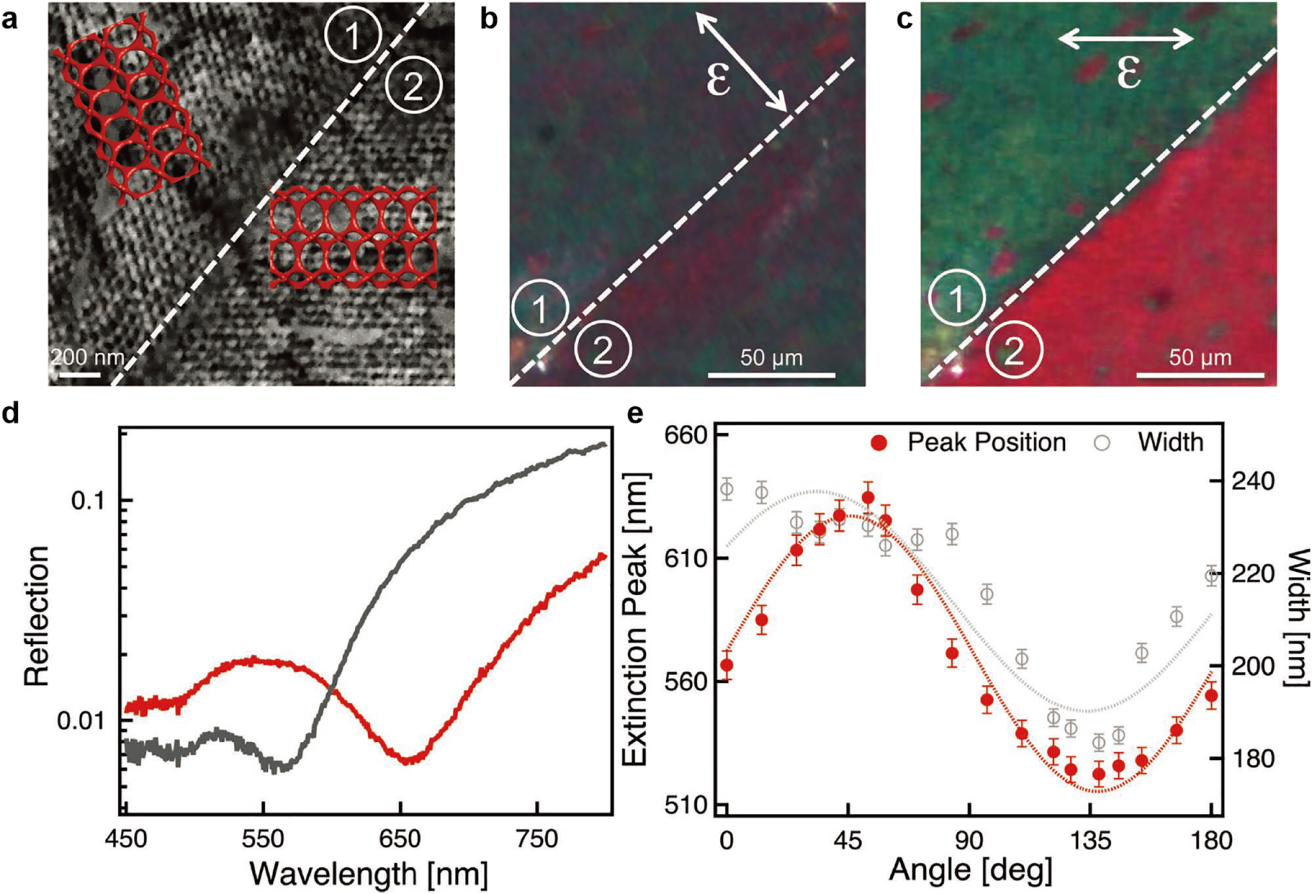
Linear dichroism of single Au-gyroid structure. (a) Scanning electron microscope (SEM) image of single Au gyroid domains with grain boundary line. (b) and (c) Opical reflection images in responses to incident polarized lights (b) perpendicular to the line and (c) parallel to [100] gyroid direction. (d) Reflectance spectra of Au-gyroid structure located at area ①, wherein polarized lights are perpendicular (red) and parallel (gray) to the [100] gyroid direction. (e) Extinction peak position and width as a function of angle between [100] gyroid direction and polarized lights. Reproduced with permission from ref. [3]. Copyright 2011, Wiley-VCH Verlag GmbH Weinheim (a)–(e).

As demonstrated in Section 3, a polymeric gyroid is required to be replaced with an optically functional element such as metals and high-refractive-index dielectric materials for optical metamaterials and photonic crystals. Using PI-*b*-PS-*b*-PEO triBCPs, Steiner et al. selectively substituted the PI channel of the PI-*b*-PS-*b*-PEO gyroid structure with Au or Ni using an electroplating process, and subsequently, eliminated the organic components [3, 4]. Using a single Au-gyroid structure with a unit cell size of 50 nm that is significantly below the visible wavelengths (Figure 6a), they observed the linear dichroism as an optical chirality of metamaterials, wherein the linearly polarized light coupled in a manner distinct to that of the localized plasmon resonances of the Au networks. Consequently, the optical reflection images (Figure 6b–c) and spectra (Figure 6d–e) revealed a birefringence across the twinned gyroid grain boundary. Alternatively, Ho et al. utilized an electroless plating method to enable the nucleation and growth of metals such as Ni, Au, and Pt in two alternating channels of the PS-*b*-poly(lactic acid) (PS-*b*-PLLA) gyroid structure [104], [105], [106]. They controlled the number of nucleation sites during the electroless plating process to selectively replace a single gyroid channel with Ni [107]. Generally, inorganic or hybrid BCP gyroid structures have been designed for various applications such as hybrid solar cells, antireflective coatings, electrochromism, photocatalysts, photonic crystals, and optoelectronics [108], [109], [110], [111], [112], [113]. In contrast, the nanophotonic applications of the BCP-templated inorganic gyroids have been limited to such chiral metamaterials. This is because the accessible unit-cell size of the BCP-templated inorganic gyroids was generally less than 100 nm. In context, larger unit-cell sizes of 200–250 nm are required to expand the window of nanophotonic applications to the visible photonic crystals and Weyl materials. However, the development of this gigantic BCP gyroid structure has been challenging thus far, as discussed in Section 4.4.

### 4.3 Assembly of BCP gyroid films in controlled orientation

**Figure 7:**
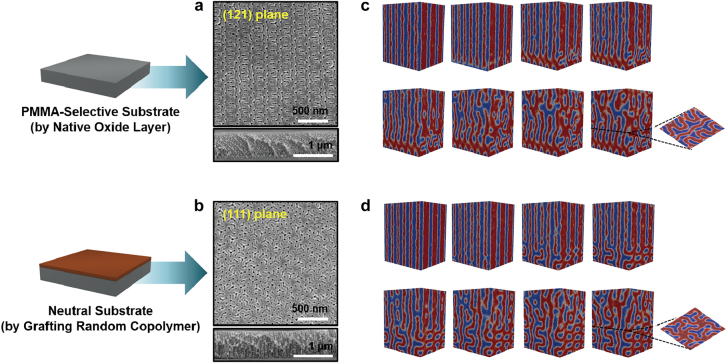
Controlled orientation of gyroid structures by modulating substrate interactions. (a) and (b) Top- and cross-sectional view SEM images of the PS-*b*-PMMA gyroid structures with (a) (121) and (b) (111) orientations. Selective and neutral interactions cause parallel and perpendicular orientations of cylinders on substrates, respectively, which consequently results in the (121) and (111) orientations of gyroid structures, respectively. (c) and (d) morphological evolution predicted in simulations of BCP films developed on (c) selective and (d) neutral substrates. Reproduced with permission from ref. [53]. Copyright 2017, American Chemical Society (a)–(d).

On the contrary, the crystal orientation of a BCP gyroid film can be controlled to surpass the interfacial interactions governing the polymeric films confined in the air/polymer and polymer/substrate interfaces. This effect could be essential in materializing the practically viable optical metamaterials, photonic crystals, and Weyl materials. A long-range-ordered gyroid structure with (121) orientation was thermally achieved from the PS-*b*-PI film supported on a preferential substrate, and the structural information of numerous gyroid planes was appropriately characterized by 2D grazing-incidence small-angle X-ray scattering (GISAXS) measurements [66]. Unlike thermally prepared gyroid films close to equilibrium, a facile approach for ordering BCP films is to utilize good solvents that vaporize and permeate the films, which is an efficient method for mediating the surface energy or interfacial interactions. Note that the solvent should be carefully selected considering the relative affinity toward each block, because the gyroid structures are formed in the swollen state, exceptionally in a narrow range of minor compositions (i.e., 0.35 < *f* < 0.39) [52]. If deviated, the BCP films are more favorable for the formation of lamellar or cylindrical morphologies. Generally, the non-preferential or neutral solvent vapor enabled the BCP chains to swell and assemble into gyroid structures with a (121) plane parallel to the film surface, wherein the transient cylinders developed from the as-cast film underwent an order-to-order transition into the gyroid structures [50, 51, 67]. Based on the epitaxial relationship between the transient cylinders and gyroid structures, Ryu et al. successfully demonstrated that large-area directed orientations of gyroid structures can be achieved from a PS-*b*-poly(methyl methacrylate) (PS-*b*-PMMA) film by modulating substrate interactions [53]. During the solvent vapor annealing (SVA) process with tetrahydrofuran, which is neutral toward the PS and PMMA blocks, the gyroid structures exhibited two distinct surface morphologies such as the (121) and (111) orientations of the gyroid films supported on the selective and neutral substrates, respectively (Figure 7a and b). These were uniformly transitioned from the parallel and perpendicular orientations of the transient cylinders, respectively. This order-to-order phase transition and gyroid orientations during the SVA process were theoretically supported by brute force Monte Carlo simulations with a coarse-grained model (Figure 7c and d). As a matter of fact, the lower-*q* {110} diffraction spots that would be forbidden in a cubic gyroid structure were distinctly seen in the 2D GISAXS patterns of gyroid-forming PS-*b*-PMMA (176–102 kDa) films with a (121) orientation, presumably suggesting a correlation between crystal orientation and non-affine deformation in the BCP gyroid film.

### 4.4 Assembly of giant BCP gyroid toward visible-wavelength photonic crystals

In most BCP gyroid assemblies, unit cells of sizes less than 100 nm have been realized with high frequency. However, this relatively small unit-cell size restricts the nanophotonic application of a BCP gyroid to optical metamaterials. Thus, a large unit-cell size (above 200–250 nm) of the gyroid assembly is an essential prerequisite for photonic crystals and Weyl materials, which can be enabled by increasing the available molecular weight of BCPs.

**Figure 8:**
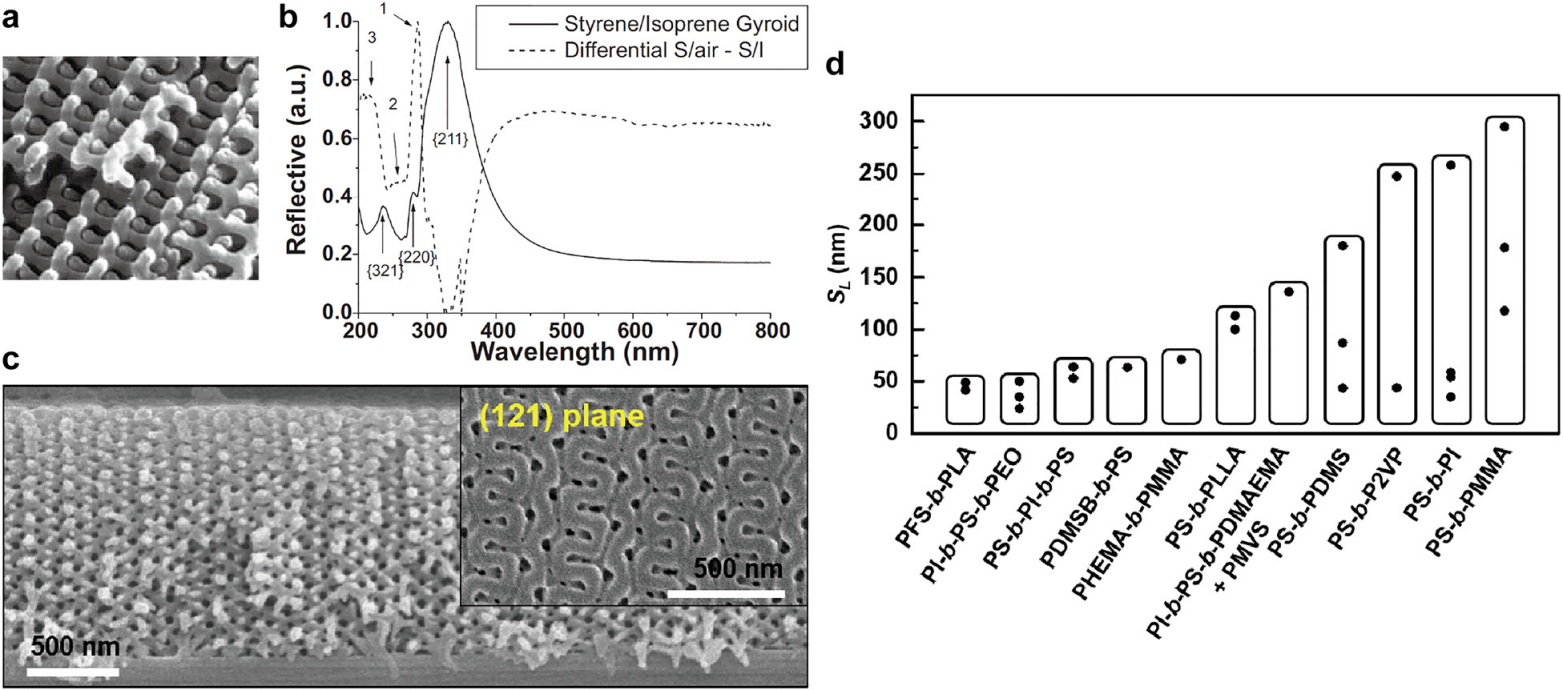
Gyroid structures obtained from solvent-assisted processes for HMW BCPs and lateral unit-cell size (*S*_L_) of various BCP gyroids. (a) SEM image of PS gyroid structure (as fracture surface), which was obtained from UV-etched PS-*b*-PI (300–450 kDa). *S*_L_ was estimated as 258 nm by *S*_L_ = $\sqrt{2}\times 2\pi /q_{\left\{220\right\}}$ . (b) Reflectance spectra of PS-*b*-PI (solid curve) and PS (dotted curve) gyroid structures. For PS-*b*-PI film, optical reflectance peaks at *λ* = 324, 281, and 212 nm correlate with {211}, {220}, and {321} planes of gyroid structures, respectively. (c) Cross-sectional SEM image of giant (*S*_L_ = 295 nm) gyroid structure and top-view image, which was obtained from 600-kDa PS-*b*-PMMA. (d) Representative gyroid *S*_L_ values reported to date. Reproduced with permission from ref. [11]. Copyright 2002, Wiley-VCH Verlag GmbH Weinheim (a) and (b); reproduced with permission from ref. [50]. Copyright 2016, Nature Portfolio (c).

Upon increasing the temperature above the glass transition temperature (*T*_g_), the translational ordering of BCP chains into assemblies can be facilitated. However, in the case of (ultra) high-molecular-weight (HMW) BCPs, thermal treatment is still insufficient for microphase separation into the desired morphology. Therefore, it is indispensable to utilize the solvent-assisted processes including the SVA and solvent casting processes, because the solvent power has been proven as sufficiently energetic for plasticizing highly entangled polymer chains. In context, Thomas et al. reported a giant PS gyroid structure (Figure 8a) that was obtained from the solvent casting of ultra HMW PS-*b*-PI (300–450 kDa) using a BCP solution in toluene and subsequent ultraviolet (UV) etching to remove the PI matrix. The lateral unit-cell size (*S*_L_) of PS-*b*-PI was estimated as 258 nm by *S*_L_ = $2\sqrt{2}\times {2\pi /q}_{\left\{220\right\}}$ . Specifically, in response to UV light, the correlation of the optical reflectance (Figure 8b) with the gyroid planes can be experimentally obtained as a consequence of the large *S*_L_ of the gyroid structure [11]. However, this *S*_L_ still restricted the photonic bandgap wavelength of the BCP gyroid to the UV regime. To leverage the working wavelength to the visible regime, a unit-cell size needs to exceed 258 nm, while it has been challenging to obtain much larger-size gyroids due to synthetic difficulty in achieving the ultra HMW BCPs.

Toward this end, Ryu et al. exploited a long-term stable SVA process with ultra HMW PS-*b*-PMMA (367–233 kDa) to achieve a giant gyroid structure, which reaches the level of *S*_L_ = 295 nm in a film geometry (Figure 8c). In particular, the top-surface (inset) SEM image corresponded to a (121) gyroid plane, and the cross-sectional view across the entire film thickness represents a large-scale interconnected morphology of a long-range-order gyroid structure [50]. As observed in the diagram for the structural sizes of polymeric gyroids (Figure 8d), a wide range of gyroid scales has been developed from various BCP self-assemblies [4, 11, 35, 37, 39, 50], [51], [52, 56, 63, 64, 68, 101], [102], [103, 107, 108, 111, 114, 115]. Overall, the size-scale of the BCPs is proportional to *χ* ^1/6^ *N* ^2/3^ in the strong-segregation limit [116], where *χ* and *N* denote to the Flory–Huggins interaction parameter and the total number of segments, respectively. The wide range of gyroid scales enables us to utilize the gyroid structures as both metamaterials and photonic crystals by means of the component substitutions for each use. Namely, small-size metallic gyroids would work as metamaterials at sub-optical wavelength scale, whereas for large-size dielectric gyroids at the optical wavelength scale of visible light, the cumulative interfacial reflections across a gyroid material elicit the photonic crystal effects associated with photonic bandgaps [72]. In this diagram, the *S*_L_ = 295 nm from HMW PS-*b*-PMMA is the largest unit-cell size among the BCP gyroids achieved to date, which may leverage the BCP gyroid nanophotonics onto the visible photonic crystals and Weyl materials.

## 5 Deformed BCP gyroid & Weyl materials

In this section, we discuss the realistic structure of the BCP gyroids based on perspectives of affine and non-affine transformations under experimental observations. The mutual complementary combinations of these two transformations can express the BCP gyroids, which can be realistically available.

### 5.1 Experimental benchmarks of lattice distortions of BCP gyroid

The self-assembled gyroid structures can be used not only for versatile template for material substitutions but also for a soft crystal that is tunable in structural periodicity and symmetry based on the soft and flexible properties of highly ordered structures. Relatively, the non-uniform lattice deformation or distortion of the gyroid structures is nontrivial in terms of the unique crystallography associated with a symmetry-breaking character. Sakurai et al. applied an external mechanical force on the gyroid structures of elastomeric PS-*b*-poly(butadiene)-*b*-PS (PS-*b*-PB-*b*-PS) and observed lower-*q* {110} and {200} reflections, which were absolutely forbidden in a cubic double-gyroid structure [60]. Gruner et al. identified the symmetry-breaking behavior in the uniaxially contracted gyroid structure of PI-*b*-PEO/aluminosilicate [61], where solvent evaporation induced uniaxial contraction to create triclinic crystallites. Notably, they initially demonstrated the concepts between the affine and non-affine deformations of the gyroid structures arising from the uniformly and nonuniformly distributed strains, respectively. With the BCP gyroid structures confined in a film geometry, Hillhouse et al. reported that the gyroid films contract in the normal direction to the substrate [62]. They reported the generation of the {110} diffraction spots in 2D GISAXS patterns as evidence for the non-affine distortion that may result in the symmetry-breaking behavior.

**Figure 9:**
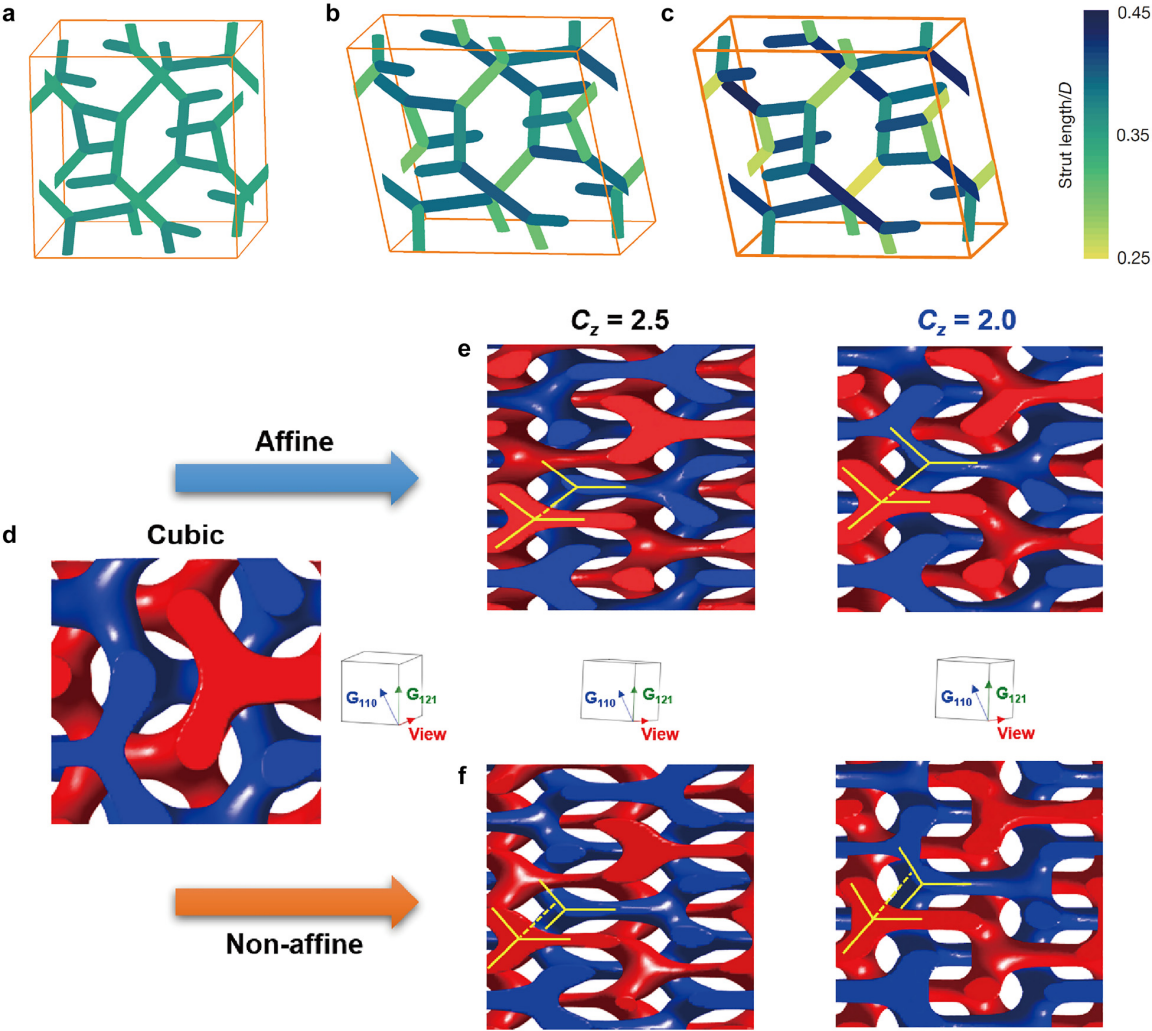
Structural characteristics of BCP gyroids in cubic, affine, and non-affine structures. (a)–(c) Skeletal graphs for (a) cubic, (b) affine, and (c) non-affine gyroid models derived from self-consistent field calculations. (d) PS-free (PMMA-channels) electron-density difference (Δ*ρ*) map of cubic double-gyroid structure. (e) Simulated affine and (f) experimental non-affine maps for gyroid structures with *z*-directional contraction ratio (*C*_*z*_) of 2.5 and 2.0. Reproduced with permission from ref. [56]. Copyright 2019, Nature Portfolio (a)–(c); reproduced with permission from ref. [35]. Copyright 2021, Elsevier B.V. (d)–(f).

Recently, Thomas et al. observed the mesoatomic distortion of the soft gyroid structure in PS-*b*-poly(dimethylsiloxane) (PS-*b*-PDMS) using a 3D tomographic imaging technique [56]. In correlation to the symmetry-breaking character, the gyroid struts in a triclinic unit cell were not distorted in an affine manner, but rather distorted in a non-affine fashion, so that the struts can accommodate deformations at the invariant angles (Figure 9a–c). For a non-affine peculiarity of noncubic gyroid structures in PS-*b*-PMMA films, Ryu et al. further unveiled the electron-density difference (Δ*ρ*) map of a cubic gyroid structure (Figure 9d), simulated affine map (Figure 9e), and experimental non-affine map (Figure 9f), where the Δ*ρ* maps were extracted from the GISAXS results [35]. In the experimental non-affine map caused by non-uniform strains, the skeletal tripod (yellow) lines of the alternating (red and blue) PMMA channels were separated from each other in contrast to the concerted lines of the affine maps with uniformly distributed strains. In addition, a notable symmetry-breaking behavior in the non-affine gyroid structure elicits such forbidden {110} and {200} reflections. Similarly, understanding the gyroid deformation and distortion associated with symmetry-breaking characteristics is essential for gyroid applications such as topological photonics from the middle-UV to visible wavelength region.

In 2002, Thomas et al. numerically calculated the photonic band structures of 3D bicontinuous cubic microphases using the plane-wave expansion method to solve Maxwell’s equations [21]. Based on the given parameters of volume fraction and dielectric contrast between media, several single networks such as single primitive, single diamond, and single gyroid were found to exhibit a complete photonic bandgap, whereas the double network variants possessed no complete bandgap. Among the double network homologs, only the double gyroid structures displayed a pseudo-bandgap, especially when the density of states goes to unity for a specific frequency. In 2014, Ho et al. demonstrated the simulated optical responses to double, shifted double, and single gyroid structures using FDTD simulations [51], wherein the channel and matrix were arbitrarily replaced with TiO_2_ and air, respectively, to modulate the refractive properties of gyroid channels. Despite the drastic increase in the dielectric contrast between the channel and matrix, the double gyroid structure exhibited no photonic bandgap. However, the single gyroid structure possessed a complete photonic bandgap that enabled the reflection, regardless of the direction of its incidence. Shifting two alternating channels associated with the local distortion elicits a partial photonic bandgap, which indicated the soft-crystal photonic property in response to the structural deformation. Overall, lattice deformations have been prevalent in self-assembled BCP gyroids, and more importantly, they cause non-trivial variations in the relevant photonic properties. Thus, the quantification of lattice transformation of a gyroid crystal can play a critical role in envisioning the photonic perspectives of a BCP gyroid, as will be detailed in the following section.

### 5.2 Linear deformation (affine transformation of *f*_SG_(x))

Let us recall the established linear function *x* = *aX* + *x*_0_ (*a* > 0), where *a* and *x*_0_ are constants independent of both *X* and *x*. In general, the distance between any two points *X*_1_ and *X*_2_ is compressed or elongated by a single parameter *a*, and the deformed distance was moved by parameter *x*_0_. This is the simplest example of an affine deformation.

The affine transformation comprises both linear deformation and translation. First, a small-line segment vector *δ* **X** is considered in a gyroid structure. We can assume the following deformation that transforms this segment into *δ*x.

(29) δx=F⋅δX

Based on the chain rule δ*x*_*i*_ = (∂*x*_*i*_/∂*X*_*j*_)δ*X*_*j*_, we obtain *F*_*ij*_ = ∂*x*_*i*_/∂*X*_*j*_ or

(30) $$F=\left[\begin{array}{lll} \frac{\partial x_{1}}{\partial X_{1}} & \frac{\partial x_{1}}{\partial X_{2}} & \frac{\partial x_{1}}{\partial X_{3}}\\ \frac{\partial x_{2}}{\partial X_{1}} & \frac{\partial x_{2}}{\partial X_{2}} & \frac{\partial x_{2}}{\partial X_{3}}\\ \frac{\partial x_{3}}{\partial X_{1}} & \frac{\partial x_{3}}{\partial X_{2}} & \frac{\partial x_{3}}{\partial X_{3}} \end{array}\right]\text{.}$$

**Figure 10:**
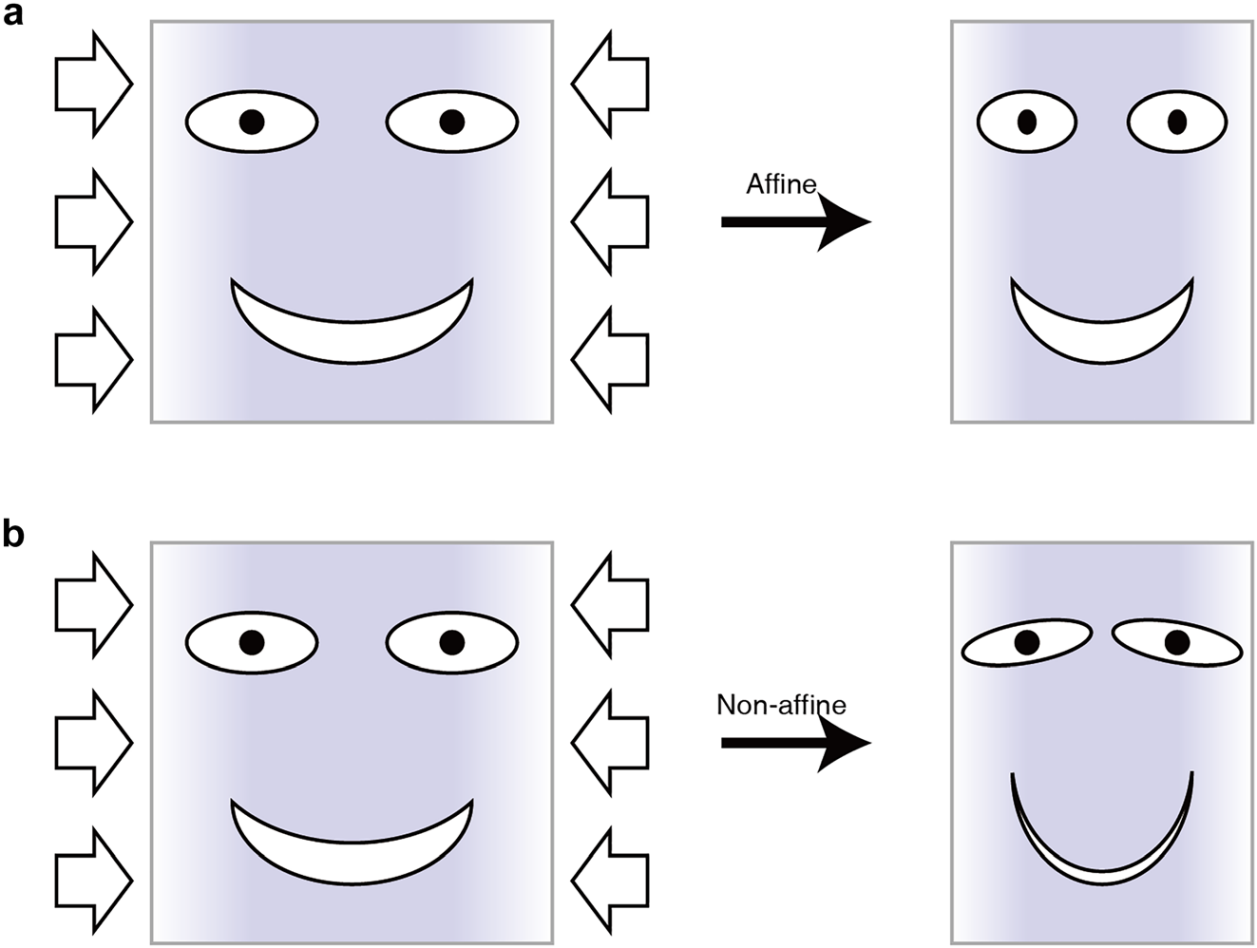
Affine and non-affine transformations. In case a structure is externally compressed, affine transformation exhibited uniform deformation proportional to the external input, whereas non-affine transformation generates position-dependent deformation.

If all the components of **F** are expressed as constants independent of both **X** and **x**, this corresponds to the situation in which all the internal points underwent uniform strain for the external deformation, which is a linear deformation, as depicted in Figure 10a.

For the equation *F*_*ij*_ = ∂*x*_*i*_/∂*X*_*j*_ with constant **F**, one of the solutions can be given by the following affine transformation:

(31) x=F⋅X,

or inversely,

(32) $$X=F^{-1}\cdot x\text{.}$$

Thus, a point **X** in a gyroid undergoes an affine transformation if the deformed point **x** is expressed as the above equation. Moreover, the invertible matrix **F** is concerned with the deformation, so called “deformation gradient.” As discussed earlier, the linear deformation is deduced from the uniform **F** distributed throughout the given material. Consequently, all the strains evaluated based on **F** become constant as well.

The lattice vectors of the primitive and conventional cells of the deformed gyroid structures, respectively, **a**_*i*_ and **s**_*i*_, are expressed as

(33) $$a_{i}=F\cdot A_{i}\text{,}$$

and

(34) $$s_{i}=F\cdot S_{i}\text{.}$$

For the deformation under **F**, the standard gyroid level-set surface (Eq. (13)) can be rewritten by substituting Eq. (32) into Eq. (13):

(35) $$f_{\text{SG}}=f_{\text{SG}}\left(F^{-1}\cdot x\right)$$

Although the uppercase and lowercase letters are used for the undeformed and deformed states, respectively, they can be used for describing the cubic and triclinic conventional gyroid unit cells as well, respectively. A majority of the BCP gyroids are generated in triclinic unit cells instead of cubic cells. Thus, we considered the lattice vectors of the cubic and triclinic cells as **S**_*i*_ and **s**_*i*_. If we aim to mathematically express a BCP gyroid in triclinic unit cells, and if the **s**_*i*_ of a triclinic cell is known, the following deformation gradient can be formulated:

(36) $$F=\frac{1}{a}\sum_{i=1}^{3}s_{i}⊗{\hat{S}}_{i}\text{,}$$

where the last vector is not **S**_*i*_ but ${\hat{S}}_{i}=S_{i}/\left|S_{i}\right|$ . Thus, the dimension of **F** is one, and this tensor transforms a variable in the length dimension to another variable in the length dimension. The vectors ${\hat{S}}_{1}$ , ${\hat{S}}_{2}$ , and ${\hat{S}}_{3}$ are arbitrary orthonormal vectors, i.e., ${\hat{S}}_{i}\cdot {\hat{S}}_{j}=\delta_{ij}$ . As several sets of ${\hat{S}}_{i}$ can be used, the level-set surface of the triclinic gyroid can be placed on various orthonormal axes sets. Moreover, the lattice constant *a* is cancelled in case the above **F** is substituted into Eq. (35). Therefore, any positive value can be temporally used for *a*.

### 5.3 Weyl photonic crystals via affine transformation

A Weyl point is a source or sink of the Berry fluxes, and it appears as a point degeneracy where two adjacent bands linearly contact in a 3D momentum space [59, 117], [118], [119]. In particular, a Weyl photonic crystal is a 3D periodic structure that exhibits Weyl points. Owing to its peculiar photonic phenomena such as the Fermi arc [117] and one-way wave propagation [79, 80], intensive research efforts have been devoted to materializing the photonic Weyl materials, including Weyl photonic crystals.

One of the necessary conditions for realizing the Weyl points includes the breaking of the inversion symmetry [28, 36, 71]. However, this condition is just the necessary condition. Although several methods exist for breaking this symmetry of the double gyroid, none of the resulting structures exhibited Weyl points. Thus, we would aim to explain this from another perspective.

In the given momentum space, each Weyl point carries a positive or negative topological charge, which is explained which can be quantitated by the Chern numbers. When the time-reversal symmetry holds, any two Weyl points with the same charges are located at the positions where inversion-symmetry is preserved with respect to the Γ point. If *N* positively charged Weyl points exist, another *N* negatively charged Weyl points should exist as well. Therefore, if the time-reversal symmetry holds, the minimum number of Weyl points is four, and they form a single plane [29].

Let us assume that these Weyl points are on the *xy*-plane in the orthotropic momentum space. As such, we can aim to clarify their existence on this plane instead of the *yz*- or *zx*-planes. This is the vital aspect for the formation of Weyl points with D_2_-symmetry of a double gyroid, as reported recently [29]. This study assumes a double gyroid comprising two single gyroids with varying refractive indices to break the inversion symmetry. When its primitive cell before deformation is defined by **A**_1_ = (*a*/2)[−1,1,1], **A**_2_ = (*a*/2)[1,−1,1], and **A**_3_ = (*a*/2)[1,1,−1], the double gyroid is deformed by

(37) $$F=\left[\begin{array}{lll} \text{cos }\theta & \text{sin }\theta & 0\\ \text{sin }\theta & \text{cos }\theta & 0\\ 0 & 0 & 1 \end{array}\right]\text{,}$$

which can be decomposed as

(38) $$F=\sum_{i=1}^{3}\lambda_{i}\left({\hat{N}}_{i}⊗{\hat{N}}_{i}\right)\text{,}$$

where the principal stretches *λ*_*i*_ are expressed as the following eigenvalues

(39) $$\left[\begin{array}{l} \lambda_{1}\\ \lambda_{2}\\ \lambda_{3} \end{array}\right]=\left[\begin{array}{l} \text{cos }\theta +\text{sin }\theta \\ \text{cos }\theta -\text{sin }\theta \\ 1 \end{array}\right]\text{,}$$

and the principal directions ${\hat{N}}_{i}$ are expressed using the following orthonormal eigenvectors

(40) $$\left[\begin{array}{l} {\hat{N}}_{1}\\ {\hat{N}}_{2}\\ {\hat{N}}_{3} \end{array}\right]=\left[\begin{array}{l} \left(1/\sqrt{2}\right)\left[1,1,0\right]\\ \left(1/\sqrt{2}\right)\left[-1,1,0\right]\\ 0,0,1 \end{array}\right]\text{.}$$

**Figure 11:**
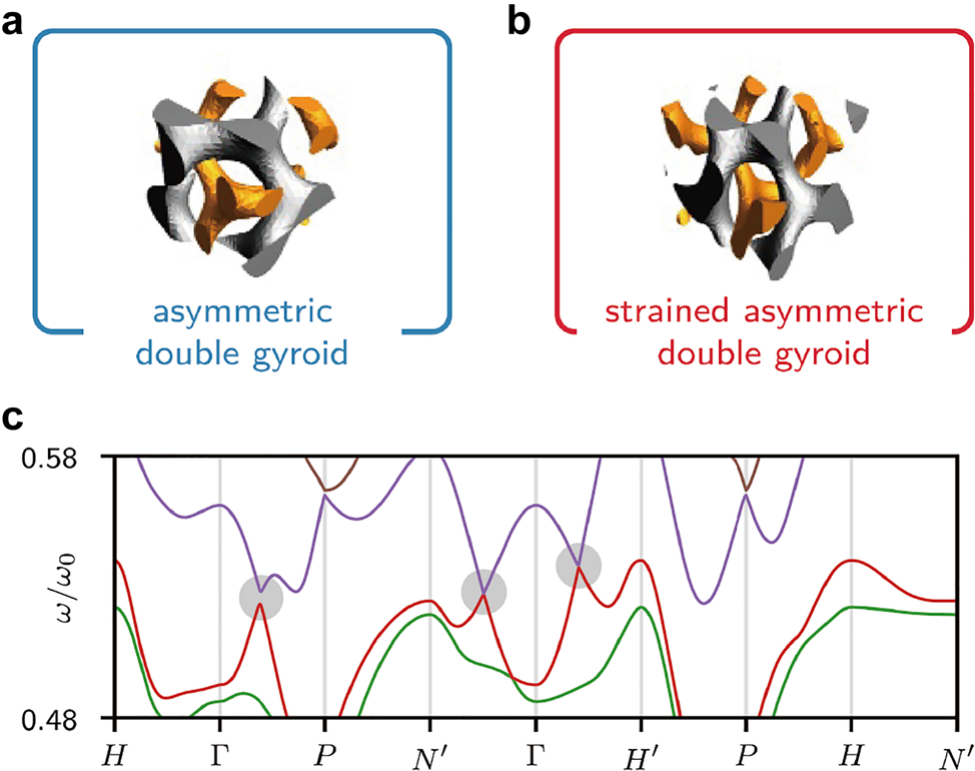
(a) and (b) Affine transformation deforming asymmetric double gyroid to strained asymmetric double gyroid. (c) Band structure exhibiting Weyl points. Reproduced with permission from ref. [29]. Copyright 2018, National Academy of Sciences (a)–(c).

For the asymmetric double gyroid (see Figure 11a), a deformed shape with a positive *θ* is depicted in Figure 11b. In fact, the general double gyroids do not show such affine transformations because the material properties (elastic moduli, Poisson’s ratio, density) of the double gyroids and the matrix materials are different in most cases. Prediction of such deformations (non-affine deformations) for the external deformation input requires numerical analysis such as finite element method based on the mechanics of materials. However, if the material properties are not so different between the double gyroid and the matrix, assuming such affine transformations is acceptable. The two major highly symmetric directions of the undeformed double gyroid are expressed as ΓN = (1/2)(−**B**_1_ + **B**_2_) and ΓH = (1/2)(**B**_1_ + **B**_2_ − **B**_3_) (as shown in Figure 3c), and their directions are the same as ${\hat{N}}_{2}$ and ${\hat{N}}_{3}$ , respectively. Thus, ΓN and ΓH are along the principal directions, and the directions $\Gamma {\text{N}}^{\prime }=\left(1/2\right)\left(-b_{1}+b_{2}\right)$ and $\Gamma {\text{H}}^{\prime }=\left(1/2\right)\left(b_{1}+b_{2}-b_{3}\right)$ remain unchanged after deformations based on Eqs. (37) and (38), respectively. The Weyl points are generated on the plane where the $\Gamma {\text{N}}^{\prime }$ and $\Gamma {\text{H}}^{\prime }$ are placed on. The negatively and positively charged Weyl points are located on the $\Gamma {\text{N}}^{\prime }$ and $\Gamma {\text{H}}^{\prime }$ lines, respectively (see Figure 11c).

### 5.4 Level-set function with additional terms (non-affine distortion of *f*_SG_(x))

In fact, the structural analyses of the BCP gyroid formation cannot be described based on the affine deformations (Eq. (36)) of the standard gyroid in Eq. (13)) only. As such, the deformation is nonlinear, which is non-uniform and position-dependent (see Figure 10b) because (i) the material properties are not uniform throughout the building blocks, (ii) the mechanical stiffness of the gyroid spatially vary from that of the base material, or (iii) defects types of yielding or fracture occur. Such deformations are termed as “non-affine distortions.” Although several studies have used the skeleton model to describe the nonlinear deformation of the gyroid, we analyzed the level-set surface and deformation gradient.

For nonlinear deformation gradient, the relationship between **X** and **x** is nonlinear, as expressed in Eq. (32); however

(41) $$X=F^{-1}\left(x\right)\text{.}$$

Upon substituting the above relation into Eq. (13) and collecting only the low-order terms, a set of variables is obtained, which is different from those presented in Tables 3 or 4. In particular, certain zero-valued coefficients in Tables 3 or 4 will become non-zero [35], and the same coefficients will start to vary [30]. Thus, the resulting gyroid cannot be written using Eq. (13) [30, 35], such as

(42) $$\begin{array}{l} f\left(X\right)=b_{110}\text{sin}\left\{\frac{2\pi }{a}\left(X_{1}+X_{2}\right)\right\}+b_{011}\text{sin}\left\{\frac{2\pi }{a}\left(X_{2}+X_{3}\right)\right\}+b_{101}\text{sin}\left\{\frac{2\pi }{a}\left(X_{3}+X_{1}\right)\right\}\\ \;+b_{1\bar{1}0}\text{sin}\left\{\frac{2\pi }{a}\left(X_{1}-X_{2}\right)\right\}+b_{01\bar{1}}\text{sin}\left\{\frac{2\pi }{a}\left(X_{2}-X_{3}\right)\right\}+b_{\bar{1}01}\text{sin}\left\{\frac{2\pi }{a}\left(X_{3}-X_{1}\right)\right\}\text{.} \end{array}$$

Thus, one of the convenient methods involves obtaining the sinusoidal function coefficients using the electron-density map or other appropriate methods instead of using **F** = **F**(**x**) and describing the lattice system based on the linear deformation gradient. Consequently, the affine transformation and the non-affine distortion are related to the inner and outer components of the sinusoidal functions in Eq. (1), respectively.

### 5.5 Weyl photonic crystals via “non”-affine distortions

Unlike that discussed in Section 5.3, the realization of the Weyl points is discussed herein based on the non-affine distortion that generated the level-set surface of the gyroid different from *f*_SG_(**X**) in Eq. (13). The initial study of the double-gyroid Weyl photonic crystal is reported in Ref [28], wherein the two single gyroids in the double gyroid had the same refractive index of 4.0. However, only a single gyroid from the double gyroid adopted an air sphere to break the inversion symmetry of the double gyroid. With this gyroid, the Weyl points were theoretically identified in the 3D momentum space. After two years, this finding was experimentally validated by the same research group [36].

**Figure 12:**
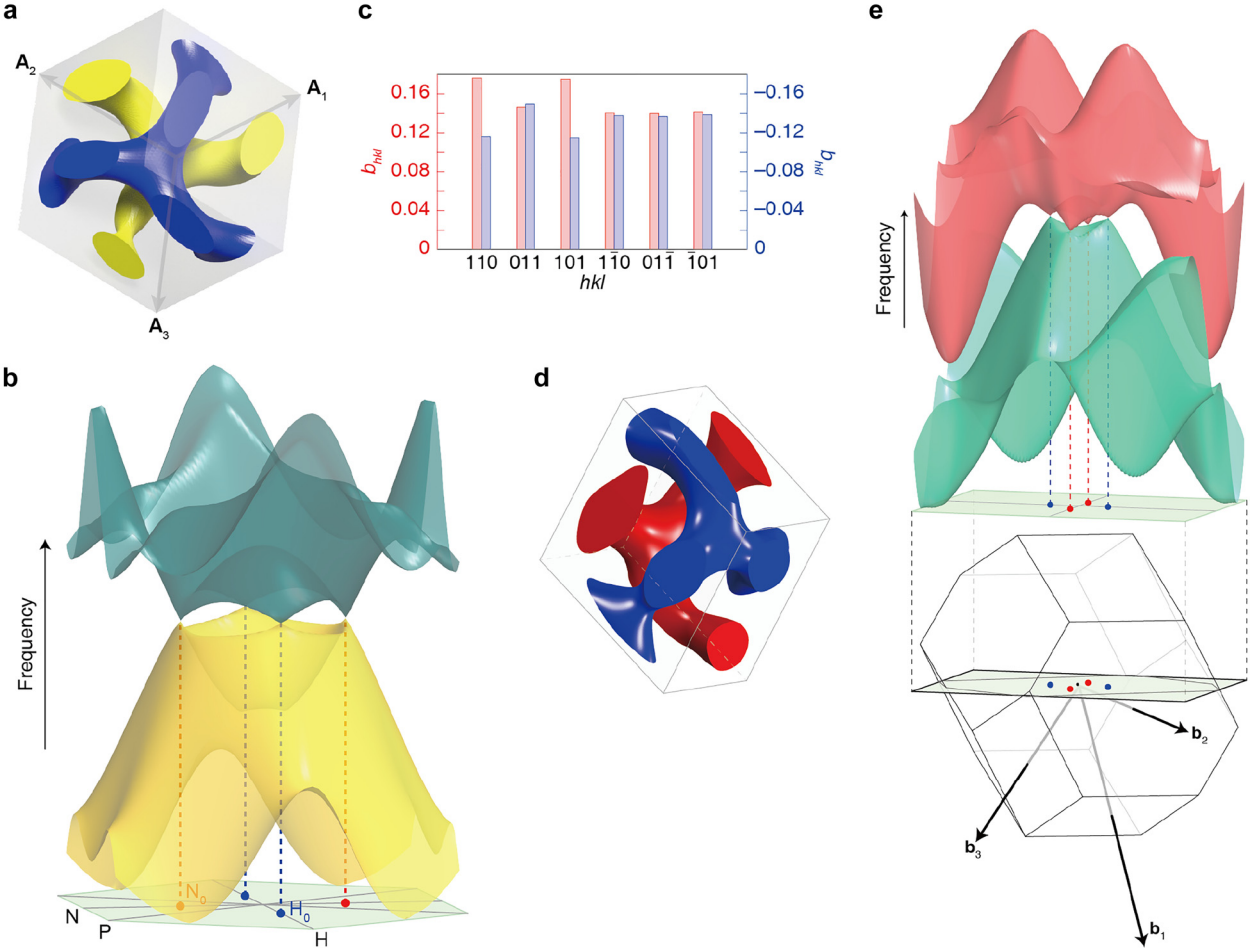
Weyl points formation via modifications of *f*_SG_(X). (a) Double gyroid comprising perturbed (yellow) and unperturbed (blue) single gyroids. Refractive indices of these are identically 4.0. (b) Photonic band structure exhibiting Weyl points. (c) and (d) Level-set analysis for two single gyroids of BCP double gyroid and its illustration. Red and blue bars in (c) detail the red and blue single gyroids in (d), respectively. The six *b*_*hkl*_ values for each single gyroid correspond to the coefficients of Eq. (42). (e) Weyl points in photonic band structure of double gyroid expressed by (c) and (d). This numerical computation considered that refractive indices of single gyroids in (d) are both 4.0. Structure was assumed as the deformed double gyroid. Reproduced with permission from ref. [30]. Copyright 2020, American Chemical Society (a) and (b); reproduced with permission from ref. [35]. Copyright 2021, Elsevier B.V. (c)–(e).

Meanwhile, such a defective double gyroid can be obtained by applying the non-affine distortion to only a single gyroid expressed under Eq. (13), as follows [30]:

(43) $$f\left(X\right)=f_{\text{SG}}\left(X\right)+p\text{ sin}\left\{\frac{2\pi }{a}\left(X_{1}+X_{2}\right)\right\}\text{,}$$

where *p* > 0 indicates the perturbation strength. The level-set surface of the counterpart single gyroid was fixed as *g*(**X**) = −*f*_SG_(**X**), satisfying O-symmetry. The single gyroids following *f*(**X**) > *f*_D2_ > 1.15 and *g*(**X**) > *f*_O_ = 1.1 form a double gyroid, as depicted in Figure 12a. Particularly, Lee’s group recently conceived perturbation strength *p* in such level-set equation; for *p* > 0, one of three arm in SG can be necked (yellow SG in Figure 12a); consequently, transforming O-symmetric SG to D_2_-symmetric SG. This double gyroid regulated by *p* > 0 also was found to potentially exhibit the Weyl points, as presented in Figure 12b. Although all these methods can exhibit frequency-isolated Weyl points, the air sphere or perturbation, which was theoretically used for inversion-symmetry-breaking, cannot be deterministically applied to a BCP double gyroid.

However, Ref. [35] proved that realistically accessible non-affine distortions of BCP gyroid could meet the structural requirement for exhibiting a Weyl point. The experimentally fabricated BCP exhibited a non-centrosymmetric distribution of *b*_*hkl*_ of the two single gyroids, according to Eq. (42) (see Figure 12c–d). Replacing the BCP as a high-refractive-index material and deforming the triclinic cell to a structure similar to the cubic cell (see Figure 12e) can be considered as the remained tasks for the experimental observation of the Weyl points.

## 6 Comparison with competing method (IL)

**Figure 13:**
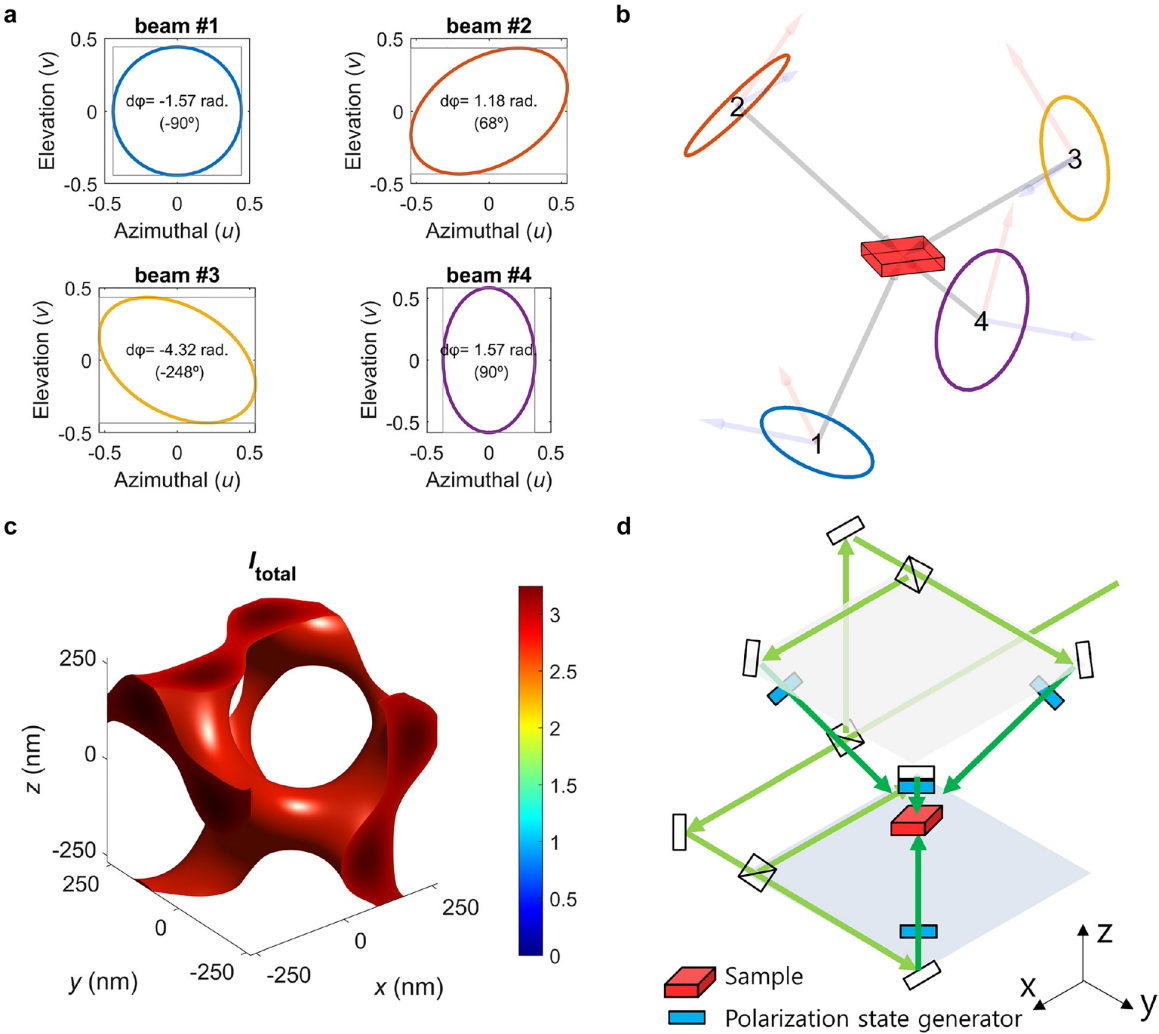
Gyroid structure realized with free-space holographic laser interference lithography. (a) Required polarization ellipses in local coordinate system of each beam, (b) overall scheme of exposure with polarization ellipses, beam propagation directions, and local coordinate systems, (c) analytically calculated result of superposition of four light beams, and (d) schematic of experimental setup for four coherent-beam generation.

To emphasize the outlook of the BCP approach, the state-of-the-art competing technologies should be exploited. In context, IL could be a promising candidate for fabricating optical single- or double-gyroids with a periodicity of approximately one micron [30]. When more than two coherent beams interfere, constructive and destructive interference of light results in a static standing periodic light wave with a periodicity comparable to that of the incident light. Moreover, IL is a nanoscopic patterning process in which the interference pattern is recorded in a photosensitive material (typically SU-8 photoresist) [22, 24, 45, 48, 49, 75, 76, 144], [145], [146]. Thus, achieving a fine pattern requires a high-quality laser along with a temperature-controlled and vibration-free setup to preserve the spatial and temporal coherency of light for preventing blurring of the pattern. Additionally, simultaneous illumination of the photoresist with more than four coherent, non-coaxial beams of light is required for 3D photonic crystals such as single-gyroids (Figure 13). The combination of light polarization determines the final structure (Figure 13a and b). Therefore, the polarization should be precisely controlled to yield an exact pattern. Using a homemade analytical code [76], the superposition of the four beams was evaluated to predict the single-gyroid structure, as shown in Figure 13c (a newly obtained result for this review).

In the post-exposure bake step, the latent image formed during the exposure turns into a chemically stable polymer, followed by the development process (selective etching by solvent) to remove the non-crosslinked portion of the photoresist. Note that capillary forces and adhesion of the structure to a rigid substrate during drying of solvent can introduce nonaffine deformations. However, this technical problem could be addressed by supercritical drying [139, 140].

To generate double-gyroid structures, two-step sequential exposures are required: the first step generates a single gyroid, while the second step generates the complementary gyroid. Furthermore, the polarization and phase of each beam should be precisely controlled between the sequential exposures. Most importantly, the alignment of the setup should be preserved throughout the experiment. As the alignment may be accidentally hampered in the process of adjusting optics, an electronic control of all parts is advised, which consequently increases the overall cost of the setup (Figure 13d). Thus, such multiple beam mixing and lithographic use are not robust enough against the mechanical vibrations, air flow, and molecular stochasticity, which are all prevalent in the common bench-top conditions and even in cleanroom conditions. This critical problem could become elucidated for using a higher number of beams to be interfered. Indeed, a nanoscale gyroid has never been achieved with a multibeam-mixing-approached IL.

**Figure 14:**
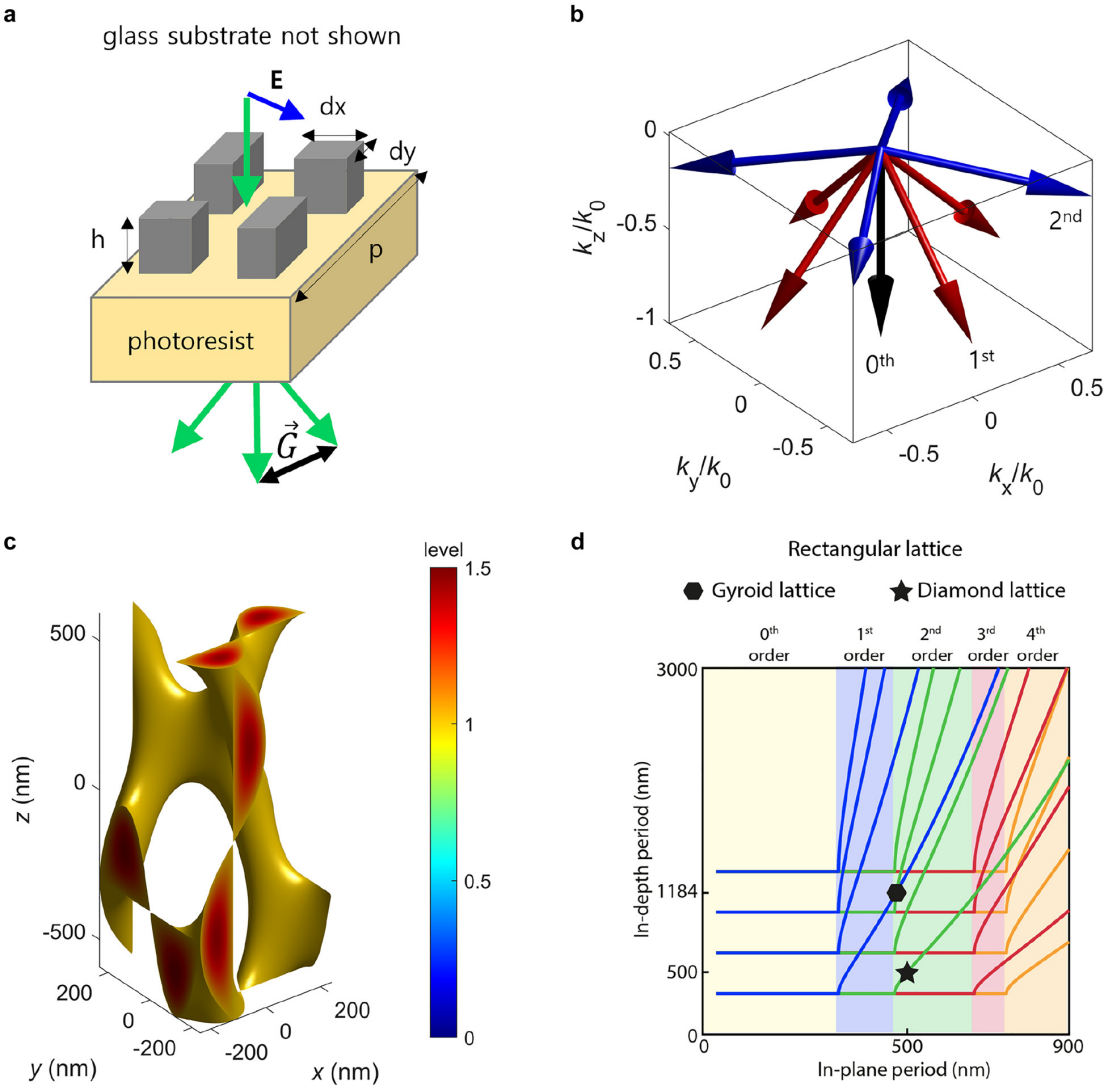
Gyroid structure realized with metasurface mask holographic interference lithography. (a) Scheme of metasurface mask and resulting grating vector, (b) diffracted beams of light from metasurface mask, generating single gyroid structure, (c) elongated single gyroid calculated with level-set equation, (d) relation of in-plane and in-depth periodicity. Reproduced with permission from ref. [146]. Copyright 2019, National Academy of Sciences (d).

Interestingly, metasurface-IL has been conceived to overcome this issue of free-space IL [146]. Metasurface consists of the multiply scaled and oriented subwavelength optical antennas (also called meta-atoms), which are quasi-periodically arrayed over the large area (retaining a supercell). According to the on-demand purpose, metasurface can be arbitrarily designed to spatially modulate the phase and amplitude of the incoming light [146]. Thus, the light passing throughout the rationally designed metasurface can be splitted into the nearly desired numbers of beams with precisely controlled intensities, polarizations, and wavevectors. After the metasurface mask is in contact with the photoresist surface, merely normal illumination of a single laser beam is enough to generate the multiple beams to interfere (Figure 14a). This is because the mask consists of a periodic supercell of the meta-atom unit cells, which acts as a 2D transmissive grating (Figure 14b). Thus, generating multiply coherent beams in the photoresist can be possible by a simple illumination of a single beam throughout the metasurface; the metasurface-IL can avoid the reliance on the direct mixing of the multibeam.

In particular, upon normal incidence throughout metasurface, the diffraction angle of light can be expressed as

(44) $$\theta_{m}={\text{sin}}^{-1}\left(1-\frac{m\lambda }{d}\right)\text{,}$$

where *m*, *λ*, and *d* denote the diffraction order, wavelength, and periodicity of the metasurface, respectively. As the mask is not required to move during the exposure and no air is present in the light path, it is immune to vibration and air turbulence effects. In addition, it does not require a complex and bulky optics setup for generating multiple beams. The remainder of the fabrication process is the same as that of the free-space IL method.

Actually, a perfect single-gyroid pattern was found to be possibly achieved from the metasurface-mask (Figure 14c). However, the reported structure is anisotropic (not cubic), for instance, elongated in the in-depth direction (Figure 14c). This single gyroid structure was realized by interfering nine diffracted beams: 0^th^ order, four 1st order (±1, 0)/(0, ±1), and four 2nd order (±1, ±1) diffraction beams. The interference between the 0^th^ order beam and the *n*th-order beam creates an in-depth periodic pattern with its own periodicity that can be determined based on the angle between the beams. The grating vector formed by the interaction of the two wavevectors is depicted by black arrows in Figure 14b. Moreover, the in-depth periodicities of all interference patterns must be satisfied to obtain a completely periodic pattern. This protocol can be performed by carefully selecting the in-plane period and the following diffraction angles (Figure 14d). The grating equation predicted that the diffraction angles of all diffraction orders are strictly linked together and not independent of each other. Therefore, it is impossible to independently control each angle. This case limits only certain potential solution candidates, which is denoted as the intersection of lines in the figure. The reported single gyroid-like structure was obtained when the in-plane period was 500 nm with a corresponding in-depth period of 1184 nm, which resulted in an elongated gyroid with an aspect ratio of greater than two. Despite this perspective, a cubic single-gyroid structure has not been realized with a metasurface-IL to date.

The following reason can serve to the additional barriers for achieving a double gyroid with the metasurface-mask technique. Based on the harmonic-nature of electromagnetic wave, a periodic surface defined with a spatial-harmonic level-set equation is compatible with the IL; for instance, a collective set of gyroid, perturbed gyroid [30], diamond, rotated cubic, and p-surface, can be fabricated using a multi-beam IL, as proven numerically and experimentally [22, 24, 45, 48, 49, 75, 144], [145], [146]. However, a reported level-set equation defining a double-gyroid structure cannot be conveniently derived. Thus, an alternative route for achieving double-gyroid structure could include the sequential exposure of complementary single gyroids with two different metasurface masks. Unless actively switching the mask such as controlling the effective refractive index of the meta-atom, the mask should be physically replaced with a new one. In this process, the precise alignment of the mask is mandatory with the overlay accuracy far under the in-plane periodicity of the pattern, which yields the IL more unrealistic for the development of a double gyroid.

Also, metasurface can be generally developed by a serial fabrication method such as EBL, because it consists of the each differently sized and oriented optical nanoantennas. Even if the required scales of the metasurfaces (e.g., lattice constant of 250 nm; lateral feature size of 60–180 nm both for 514 nm wavelength laser source-based metasurface-IL) [146] can be readily fulfilled by a common EBL, the fabrication of metasurface should still depend on the state-of-the art infrastructure, which could further hinder the democratization of metasurface-IL.

## 7 Conclusion & outlook

### 7.1 Practical limitations and perspectives of BCP optical metamaterials

**Figure 15:**
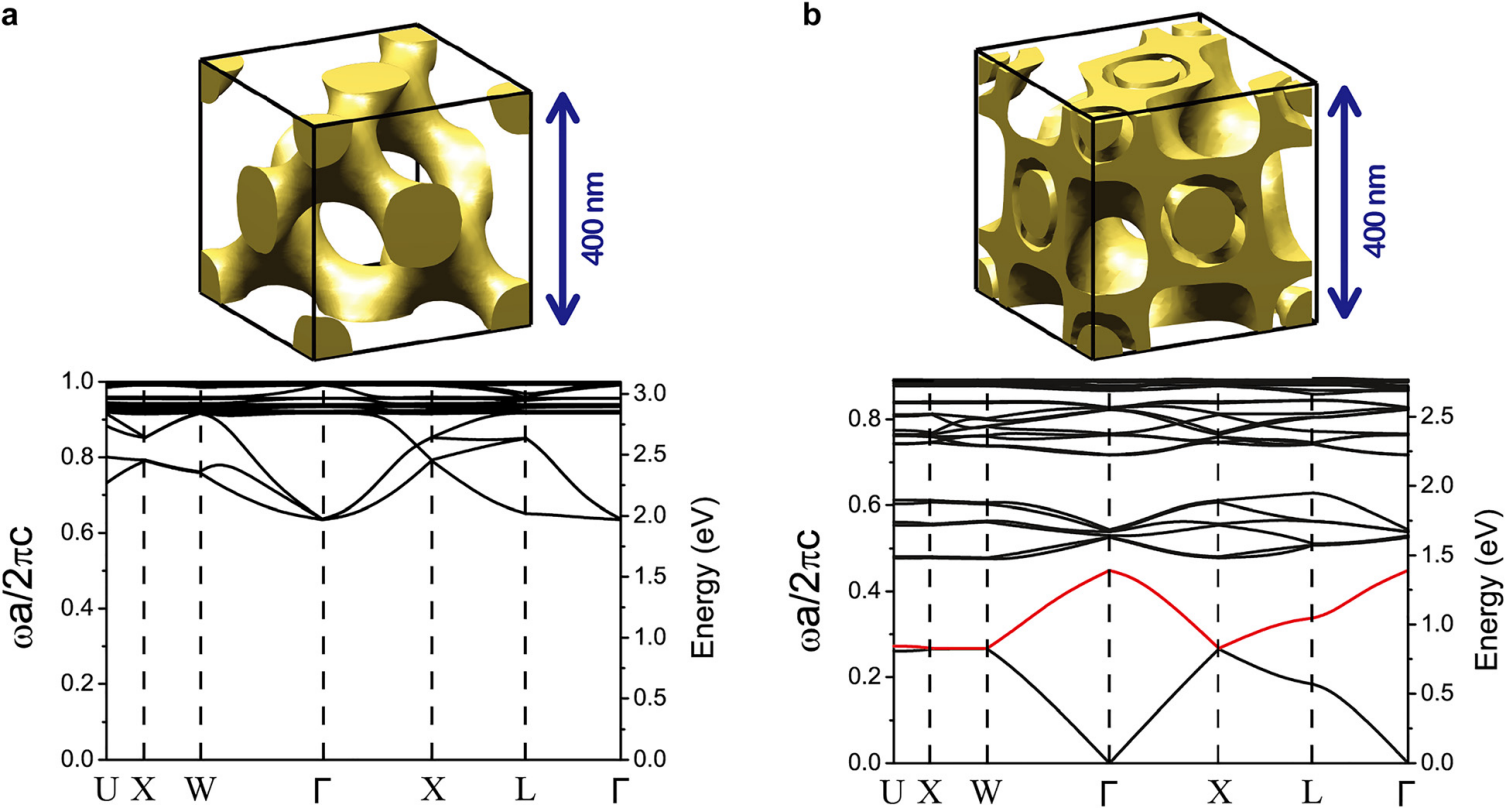
Photonic band structures of 3D continuous diamond cubic metamaterials, space group Q^227^ ( $Fd\bar{3}m$ ). (a) Metamaterial with no internal substructure and (b) metamaterial with a metal/insulator/metal coaxial substructure. Red line represents negative refraction band.

Although the BCP self-assembly is appealing as the easy-to-craft nanoscale gyroids with long-range order, the resultant assemblies are structurally error-prone to grain boundaries and defects (e.g., dislocation and disclination). However, all structural imperfections are not problematic, especially for optical metamaterials. The photonic band structures of a single diamond network is presented in Figure 15, as discussed in Section 2.3, including its two-network MIM waveguide counterpart, which has been newly obtained for this review. The single diamond structure was obtained using the level-set function:

(45) $$\begin{array}{l} f\left(X\right)=\text{sin}\left(\frac{2\pi }{a}X_{1}\right)\text{sin}\left(\frac{2\pi }{a}X_{2}\right)\text{sin}\left(\frac{2\pi }{a}X_{3}\right)+\text{sin}\left(\frac{2\pi }{a}X_{1}\right)\text{cos}\left(\frac{2\pi }{a}X_{2}\right)\text{cos}\left(\frac{2\pi }{a}X_{3}\right)\\ \;+\text{cos}\left(\frac{2\pi }{a}X_{1}\right)\text{sin}\left(\frac{2\pi }{a}X_{2}\right)\text{cos}\left(\frac{2\pi }{a}X_{3}\right)+\text{cos}\left(\frac{2\pi }{a}X_{1}\right)\text{cos}\left(\frac{2\pi }{a}X_{2}\right)\text{sin}\left(\frac{2\pi }{a}X_{3}\right) \end{array}$$

As expected, the metallic bandgap of the single diamond metamaterial was similar to that of the single gyroid counterpart (Figure 15a). However, the single diamond with MIM motif exhibited a linear dispersion negative band, as depicted using the red curve in Figure 15b. Moreover, the single-diamond structure has mirror symmetry. Thus, we can conclude that the negative refraction in the 3D continuous metamaterials did not originate from the structural uniqueness of gyroids, such as chirality, but emerged from the MIM and its waveguide mode. Therefore, the negative refractions are presumably immune to global deformations such as affine or non-affine deformation if long-range translational order is preserved, and the two bicontinuous networks are separated by an insulating layer.

Nonetheless, there are critical deformations that make negative refractions difficult to realize such as interconnections between separate metal networks or disconnections of networks, since the coupled resonances in the MIM waveguide disappear in such cases.

**Figure 16:**
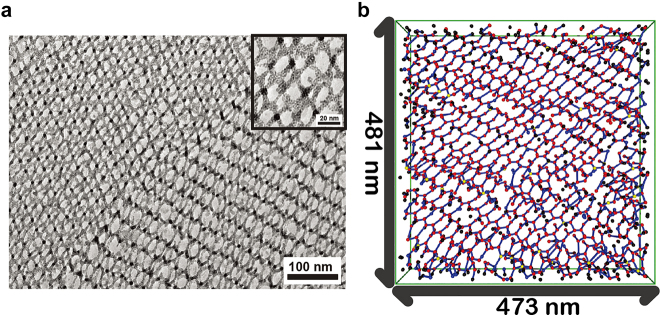
Single gyroid materials prepared by BCP/Nanoparticle co-assembly. (a) Transmission electron microscopy (TEM) image of Pt nanoparticles directed by BCP-derived gyroid structure. (b) Reconstructed single gyroid networks using TEM tomography technique. Reproduced with permission from ref. [54]. Copyright 2014, Nature Portfolio.

The defects mediating the dislocations, disclinations, and disconnections of the two networks are expected to create challenges in the experimental realization. These problems have been elucidated, especially for the sub-50-nm *S*_L_ of a BCP gyroid. For instance, Li et al. fabricated the Pt nanoparticle 3D networks directed by BCP/nanoparticle co-assembly and displayed detailed network connectivity using TEM tomography technique [54]. The network disconnections in this BCP gyroid were visible with an *S*_L_ of ∼20 nm (Figure 16a), which was further supported by the numerical simulations (Figure 16b). These structural defects could pose a critical problem in realizing gyroid optical metamaterials for optical negative refraction. As analyzed earlier, the efficient MIM plasmonic waveguide mode in the long-range order will define whether the optical negative refraction will be observed from a metallic gyroid.

The use of HMW BCPs can improve the lattice connectivity of a BCP gyroid [11, 50]. However, in this case, the working wavelength of the gyroid could be inevitably redshifted to near-infrared (NIR). Note that this red-shift of the working wavelength to the NIR regime could weaken the rationale of a BCP gyroid metamaterial (i.e., achieving a negative index at the visible), because conventional monolithic lithography has already addressed the negative-index optical metamaterials operating in the NIR, as pioneered by Zhang et al. In addition, Steiner et al. reported that the electroplating process and the subsequent elimination of the polymer components [3, 4] can be used for the highly connected metallic double gyroid. Also note that the double gyroid assembled by di-BCP is prone to the interconnections or bridging between networks at the grain boundaries, so as to ruin the possibility of negative index. However, tri-BCP gyroid allows us to avoid this interconnection between networks because it forms the alternating networks.

In contrast, chiral optical metamaterials (i.e., metallic gyroids) can be more robust against the structural defects than negative-index metamaterials. As they rely on the overall chiral geometry of deep-subwavelength-scale gyroid units, local structural defects such as dislocation and disconnectivity do not pose major influence on the ensemble of the chiral responses (according to the effective medium theory). This is the fundamental reason that a BCP gyroid was readily translated into the realizations of chiral optical metamaterials. Indeed, the measured chiro-optical response from the metallized BCP gyroid matched well with the numerical predictions [3], [4], [5, 55]. Nevertheless, it is still important to note that the relation between optical chirality and the handedness of local helices is very important [7]. In general, the optical chirality has been analyzed with the local geometry of a single gyroid [7]. As such, the sign of optical chirality (e.g., circular dichroism index) would be reversed, when the handedness of local geometry (the sign of chirality order parameter in [70]) is reversed. This is because the optical chirality of the three lowest bands depends on which helices are activated for the propagation direction and the polarization of the excitation. Therefore, the relatively large distributions of the dihedral angle in a BCP chiral optical metamaterials can cause the distributed sign of optical chirality.

### 7.2 Long-way-to-go for BCP Weyl materials

Although the BCP gyroid could potentially provide the platform for a Weyl photonic crystal as detailed in the previous section, its materialization is yet to be proven. Several issues in thee BCP self-assembly are required to be addressed. As demonstrated in Ref. [30], the topological photonic band structure of a double gyroid can drastically vary, even for a tiny structural variation. For instance, a slight variation in the perturbation strength *p* of a gyroid could ruin the accidentally optimized Weyl point. Thus, the lattice deformations of a BCP gyroid must be uniformly conducted over a large area to reliably achieve a Weyl point at optical frequencies. This implies that the uniform assembly of a BCP gyroid within a domain should be considered as a prerequisite for this purpose.

To overcome the limitations caused by grain boundaries and defects, Aissou et al. employed a trenched line pattern developed by IL, wherein the gyroid-forming BCP films were subjected to the SVA process under topographic confinement to guide the lateral alignment of the gyroid arrays and yielded larger grains (>10 μm^2^) [63]. Alternatively, Wiesner et al. introduced defect-free single-grain crystals of mesoscale gyroid structures, which were prepared by solvent evaporation-induced crystallization with a triBCP/homopolymer mixture [115]. Nevertheless, a universal method allowing for the wide-scale assembly of a single crystalline BCP gyroid is yet to be developed, regardless of the molecular weight and polymeric composition. Bates et al. reported that the epitaxial transition of the self-assembled hexagonal cylinders to the gyroid can be induced by an order-to-order transition in BCP melts [64, 65]. This finding could inspire a strategy for addressing the structural defects of a BCP gyroid. For instance, circle arrays can be first defined using electron-beam lithography (EBL), wherein their lattice should be commensurated with that of a BCP gyroid. In particular, a gigantic BCP gyroid with a molecular weight over (367–233 kDa) can satisfy the lattice size, which can be defined by the commonly accessible EBL at the university level. Thereafter, we can induce the assembly of the hexagonal cylindrical arrays and their subsequent transition into a single crystalline gyroid, which will be separately reported by our group.

An equally important issue for the optical Weyl material pertains to their ability to deterministically transform the gyroid lattice. As discussed in Section 5.3, the pioneering study on BCP gyroid Weyl materials [29] suggested an affine-deformation-based route for breaking inversion-symmetry (parity-broken, triclinic cell). However, such assembly of the single-crystalline cubic BCP gyroid and subsequent on-demand affine-deformation are not currently available, because the effective stress, which is induced during thermal- and solvent-assisted assembly of BCP (i.e., conventional assembly methods), can be differentiated according to the molecular weight of the BCP used, chemical composition of BCP blocks, target film thickness, and possible *S*_L_. Thereby, this realistic limitation restricts the BCP gyroid Weyl materials to theoretical exploitations.

Furthermore, we want to remark on the non-affine distortion. A defect-like Eq. (43) hinders the formation of a wide bandgap from a single gyroid [30]. Interestingly, such defects are crucial factors in developing double-gyroid topological photonic crystals [30]. Then, the key question arises: can it be deterministically adjusted for on-demand purposes? As recently unveiled, the non-affine distortion to triclinic cells is prevalent in a practical BCP self-assembly of a gyroid (see Sections 4 and 5). This implies that shear stress is effectively induced during the assembly process rather than normal stress, while the resultant stain is inconsistent across the gyroid crystals owing to the varying moduli of each BCP block. Therefore, a BCP double gyroid whose single gyroids are selectively asymmetric cannot be easily fabricated. Recently, Lee et al. reported that a double gyroid consisting of inversion-symmetry-breaking between two perturbed single gyroids can be obtained during a controlled SVA assembly of a BCP [35]. However, it is an accidentally obtained structure that is not universal.

More critically, the spatially controlled gyroid deformation can further expand the potential use of optical Weyl materials. For instance, we can induce a topologically protected, one-way surface mode from heterogeneous crystal systems, where a Weyl material can be seamlessly integrated with a photonic crystal with a complete bandgap. In this case, the light can propagate along the interface between a photonic crystal and Weyl material, and a highly efficient waveguide can be achieved in this system, regardless of the light propagating pathway [28, 30, 79, 118, 120]. Ideally, spatially controlled non-affine deformation on a uniformly assembled, cubic BCP gyroid is required for the transformative application of BCP Weyl material [29, 30].

### 7.3 Roadmap for BCP photonic crystals and Weyl materials

The vertical or shear stress can mitigate efficient epitaxial assembly of a single crystalline cubic gyroid, and an SVA method could possibly evolve toward this end. For example, the release rate of solvent could be designed to be much slower than that of currently used SVA, in that the mechanical stress would be minimized. With regard to spatially controlled deformation, the light-induced plastic deformation could be an effective solution. Chromophore-incorporated azobenzene polymers can undergo plastic deformation under light illuminations [76, 121], [122], [123], [124], [125]. This plastic deformation can be highly directional – parallel to the incident light polarization, while its degree is proportional to the light illumination time or intensity [126], [127], [128], [129], [130]. In particular, before the light illumination, the glassy azopolymer is stress free due to initially isotropic orientations of side chains and backbone. However, the azobenzene chromophores (side chains of azopolymer) tend to be aligned along the direction, which is perpendicular to the polarization of incoming light. Therefore, a relatively high stress can be induced owing to the entropic contribution. To withstand this internal stress, the glassy azopolymer can have the strong yield stress above a few tens of MPa [121].

More importantly, this unprecedented plastic deformation can be spatially controlled (occurring only in the light illumination area, whereas the remaining area remains intact) [125, 130, 131]. At the optical frequencies, both Weyl materials and photonic crystals demand a few hundred nanometer-scale periodic crystal (i.e., wavelength-scale). As such, a few micron-scale domain of Weyl materials and photonic crystals needs to be seamlessly integrated, implying that the patterned light illumination with a few micron-level spatial resolution, which could be readily accessible with a common photolithography, will be enough toward this end. Thus, the azobenzene-containing polymeric BCP gyroid and their light-induced deformation could yield possible solutions for BCP-gyroid Weyl materials operating at optical frequencies. If this experiment roadmap becomes concreted, we could expect that the BCP gyroids will advance other topological photonic crystals (e.g., nodal line (band degeneracies in 1D shape) [28, 71, 78, 132], [133], [134], [135], [136]) as well as the Weyl points (band degeneracies in 0D shape) [118, 137], [138], [139], [140], [141].

Note that a simple photonic bandgap mode of the BCP gyroid can be leveraged using this experimental roadmap. The upper limit of the BCP gyroid photonic bandgap reaches up to the UV regime. This record-high wavelength was attained from PS-*b*-PI (300–450 kDa) with *S*_L_ = 258 nm (Section 4.4) [11]. The achievable *S*_L_ was further extended to 295 nm using PS-*b*-PMMA (367–233 kDa) [50]. The *S*_L_ values of 258–295 nm could be compatible with the realizations of the visible photonic bandgap. However, these reported values of *S*_L_ were taken along the lateral dimension instead of the vertical dimension. Thus, the visible photonic crystals were not experimentally validated from the BCP gyroid, primarily owing to the volume shrinkage along the normal direction and the resulting squeezed unit-cell size along the vertical direction. Hopefully in the future, the development of a (near) cubic BCP gyroid with HMW can expand the working window of the photonic bandgap to the visible region, which expands the accessible material pallet for a 3D structural color pigment.

We also want to mention about material selection, which is one of the important factors in materialization of BCP gyroids toward a Weyl photonic crystal. A photonic crystal’s actuation wavelength range is about double of the lattice constant of the crystal. This means that, to actuate a photonic crystal in visible region, its lattice size should be within 100–300 nm. Although we suppose that a BCP gyroid is fabricated after resolving all the challenges mentioned in the previous section, many high-refractive-index materials exhibit high absorption in that region. For example, germanium possesses refractive indices of 4.0 in the higher wavelength region larger than 1000 nm, and it does not show significant absorption. However, below that wavelength, its absorption drastically increases [142]. This problem can be partially resolved by using silicon. For a wavelength higher than 500 nm, its refractive index is about 3.5 without absorption [143]. Lee et al. [30] theoretically showed that a double gyroid made of the silicon with a lattice constant of 281.5 nm can exhibit frequency-isolated Weyl points whose wavelengths are 510.7–512.0 nm.
